# Deciphering and Targeting the ESR2–miR-10a-5p–BDNF Axis in the Prefrontal Cortex: Advancing Postpartum Depression Understanding and Therapeutics

**DOI:** 10.34133/research.0537

**Published:** 2024-11-25

**Authors:** Fan Luo, Liming Liu, Mei Guo, Jiaquan Liang, Lei Chen, Xiaojie Shi, Hua Liu, Yong Cheng, Yang Du

**Affiliations:** ^1^ The Second Affiliated Hospital of Xinxiang Medical University, Xinxiang, China.; ^2^ Center on Translational Neuroscience, College of Life and Environmental Sciences, Minzu University of China, Haidian District, 100081 Beijing, China.; ^3^ Henan Key Lab of Biological Psychiatry, Xinxiang Medical University, Xinxiang, China.; ^4^ Institute of National Security, Minzu University of China, Haidian District, 100081 Beijing, China.; ^5^ NHC Key Laboratory of Birth Defect for Research and Prevention (Hunan Provincial Maternal and Child Health Care Hospital), Changsha, Hunan 410008, China.; ^6^ Henan Collaborative Innovation Center of Prevention and Treatment of Mental Disorder, Xinxiang, China.

## Abstract

Postpartum depression (PPD) represents a important emotional disorder emerging after childbirth, characterized by its complex etiology and challenging management. Despite extensive preclinical and clinical investigations underscoring the role of estrogen fluctuations and estrogen receptor genes in PPD, the precise mechanisms underpinning this condition have remained elusive. In our present study, animal behavioral studies have elucidated a tight link between the aberrant expression of ESR2, miR-10a-5p, and BDNF in the prefrontal cortex of mice exhibiting postpartum depressive-like behavior, shedding light on the potential molecular pathways involved. Integrating bioinformatics, in vivo, and cell transfection methodologies has unraveled the intricate molecular interplay between ESR2, miR-10a-5p, and BDNF. We identified ESR2 as a negative transcription factor that down-regulates miR-10a transcription, while miR-10a-5p serves as a negative regulator that suppresses BDNF expression. This molecular triad contributes to the pathogenesis of PPD by affecting synaptic plasticity, as evidenced by alterations in synapse-related proteins (e.g., SYP, SYN, and PSD95) and glutamate receptor expression. Additionally, primary neuron culture studies have confirmed the critical roles of ESR2 and miR-10a-5p in maintaining neuronal growth and morphology. Therapeutic interventions, including stereotactic and intranasal administration of antagomir or BDNF, have demonstrated significant potential in treating PPD, highlighting the therapeutic implications of targeting the negative transcriptional and regulatory interactions between ESR2, miR-10a-5p, and BDNF. Our findings endorse the hypothesis that estrogen fluctuations and estrogen receptor gene activity are pivotal stressors and risk factors for PPD, affecting central nervous system functionality and precipitating depressive behaviors postpartum.

## Introduction

Postpartum depression (PPD) is a significant mental health challenge characterized by severe depressive episodes that emerge within the first 4 weeks following childbirth. It has become an escalating public health concern, with a global prevalence rate of approximately 17%, making it a common mental health disorder among new mothers [1–3]. This increase in incidence not only poses a significant threat to maternal and child health but also impacts family dynamics and the social interactions of expectant mothers, thereby presenting a formidable challenge to both clinical practice and the broader field of scientific research [4–6].

The etiology of PPD is multifaceted, stemming from a combination of environmental stressors and genetic predispositions. Environmental factors include a range of life events, inadequate social support, histories of abuse, early adversities, strained mother–child relationships, personal and familial mental health histories, and challenges within intimate partnerships [5,7–10]. On the genetic and epigenetic front, alterations in genes involved in the metabolism, synthesis, and transport of monoamine neurotransmitters, key elements of the hypothalamic–pituitary–adrenal (HPA) axis, and the kynurenine pathway (KP), have been implicated as central contributors to PPD [4,7–9,11–13]. Despite these insights, a significant knowledge gap persists regarding the exact causes, mechanisms, and effective interventions for PPD. Furthermore, the distinction between PPD, perinatal depression, and general depression often blurs, overlooking the unique characteristics and complexities of PPD. This oversight presents substantial barriers to the development of targeted treatment and prevention strategies.

In light of these challenges, epigenetic research into the onset of PPD has emerged as a critical area of focus. Key epigenetic mechanisms under investigation include DNA methylation, the regulation of noncoding RNA, and transcription factor activity [14,15]. Although significant strides have been made in understanding the role of microRNAs (miRNAs) and transcription factors in various psychiatric disorders, including depression, the specific contributions of these factors to PPD remain underexplored. Our research zeroes in on the epigenetic landscape of genes associated with PPD, many of which have been previously implicated in general depression. This includes genes related to neurotransmission (e.g., 5-HTT [16] and OXTR [17–21]), components of the HPA axis (e.g., GR [18,22,23]), neurodevelopment (e.g., 11β-HSD-2 [22,23] and BDNF [16]), and others like HP1BP3 [24–28] and TTC9B [24–27]. Historically, the genetic and epigenetic research on PPD has prioritized the depression aspect, often at the expense of investigating the hormonal and behavioral shifts unique to the postpartum period. Additionally, there has been limited exploration into the complex interactions between various molecules involved in PPD.

Our study aims to bridge these gaps by elucidating the potential molecular and biological relationships between estrogen, its receptor ESR2, miR-10a-5p, and BDNF from an epigenetic standpoint. By doing so, we seek to unravel the possible pathogenesis of PPD. Crucially, our research also endeavors to identify effective strategies for the treatment of PPD, leveraging our findings to advance both our understanding and management of this complex condition.

## Results

### Bioinformatics and experimental exploration of key molecular determinants in PPD pathogenesis

**Fig. 1.**
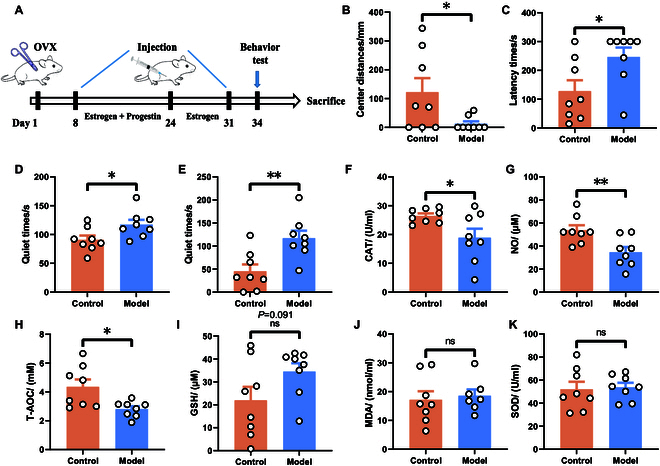
Model establishment, behavioral test, and serum oxidative stress test of PPD. (A) Establish a model of PPD. Behavioral test: (B) Distance the mice traveled in the central region of the open field. (C) Latency time of the mice for their first feeding. (D) Quiet time for mice to undergo forced swimming tests. (E) Quiet time of mice to undergo tail suspension test. Serum oxidative stress test: (F) CAT content in serum of mice. (G) NO content in serum of mice. (H) T-AOC content in serum of mice. (I) GSH content in serum of mice. (J) MDA content in serum of mice. (K) SOD content in serum of mice. Statistical analysis was performed by Student’s *t* test. *P < 0.05, **P < 0.01, ***P < 0.001 (*n* = 8).

**Fig. 2.**
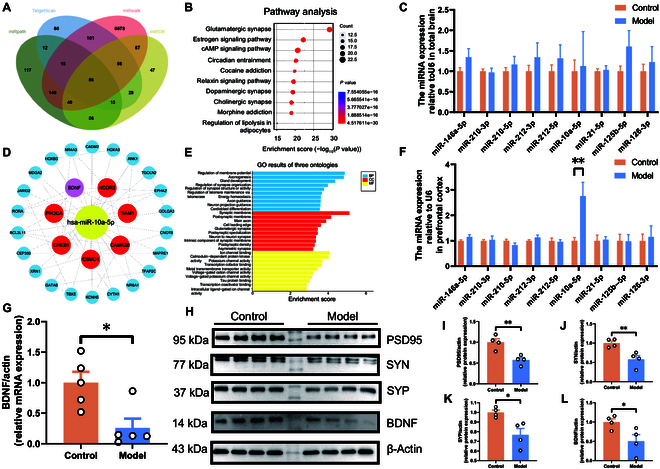
Bioinformatics analysis of target genes of miR-10a-5p and expression of synaptic related genes. (A) Venn map screen for target genes of miR-10a-5p. (B) KEGG analyses of target genes. (C) Expression of miRNAs that may be associated with PPD in the whole brain of mice (*n* = 5). (D) Interaction between target genes. (E) GO terms for target genes. (F) Expression of miRNAs that may be associated with PPD in the prefrontal cortex of mice (*n* = 5). (G) Relative mRNA expression of BDNF from the prefrontal cortex. (H) Protein expression of PSD95, SYN, SYP, and BDNF from the prefrontal cortex (*n* = 4). (I) Protein expression of PSD95. (J) Protein expression of SYN. (K) Protein expression of SYP. (L) Protein expression of BDNF. Statistical analysis was performed by Student’s *t* test. *P < 0.05, **P < 0.01, ***P < 0.001.

The establishment of the PPD mouse model followed the depicted procedure in Fig. 1A. Behavioral testing validated this model, with significant findings illustrated in Fig. 1B to E. Specifically, the ovariectomy intervention group exhibited depression-like behavior, substantiating the model’s credibility. Serum assays from eyeball blood samples, analyzed using oxidative stress kits, demonstrated a marked reduction in catalase (CAT), nitric oxide (NO), and total antioxidant capacity (T-AOC) within the model group relative to controls (Fig. 1F to H). However, levels of reduced glutathione (GSH), malondialdehyde (MDA), and superoxide dismutase (SOD) remained unchanged between groups (Fig. 1I to K), suggesting a significant antioxidant capability reduction in mice with PPD. These observations align with previous depression studies, further confirming our model’s validity. Subsequent gene identification through GeneCards (https://www.genecards.org/), DisGeNET (https://www.disgenet.org/), and Diseases (http://diseases.jensenlab.org/) databases revealed 21 common genes (search term: “postpartum depression”), including BDNF, as pivotal (Fig. S1A). Protein–protein interaction (PPI) network analysis, facilitated by the STRING (https://cn.string-db.org/) database and visualized via Cytoscape software, highlighted these proteins’ interactions (Fig. S1B). Further Gene Ontology (GO) and Kyoto Encyclopedia of Genes and Genomes (KEGG) analyses of these key genes suggested significant enrichment in pathways related to glutamatergic synapse and estrogen signaling (Fig. S1C to F). The extraction of several miRNAs, notably miR-10a-5p, from the GeneCards database was linked to PPD. Quantitative polymerase chain reaction (qPCR) analysis was conducted on tissues from both the whole brain and the prefrontal cortex. The results revealed that miR-10a-5p was significantly up-regulated only in the prefrontal cortex of the model group, with no notable changes observed in the whole brain (Fig. 2C and F). We searched the miRNA-gene databases (search term: “miR-10a-5p”): TargetScan (https://www.targetscan.org/), miRPath (https://dianalab.e-ce.uth.gr/html/mirpathv3/index.php?r=mirpath), miRDB (https://mirdb.org/), and miRWalk (http://mirwalk.umm.uni-heidelberg.de/), and the results were analyzed, ultimately identifying 56 overlapping target genes, including BDNF (Fig. 2A), with PPI network analysis revealing 29 key targets (Fig. 2D). GO and KEGG analyses underscored the involvement of these genes in synaptic connectivity and neuronal interaction (Fig. 2E), with BDNF ranking as a primary focus due to its neurogenic and synaptic roles (Fig. 2G, H, and L). Moreover, Western blot analyses confirmed the significant down-regulation of synaptic proteins PSD95, synapsin, and synaptophysin in the model group (Fig. 2H to K), implicating synaptic protein expression aberrations in PPD development.

### MiR-10a-5p’s inhibitory role on BDNF expression

**Fig. 3.**
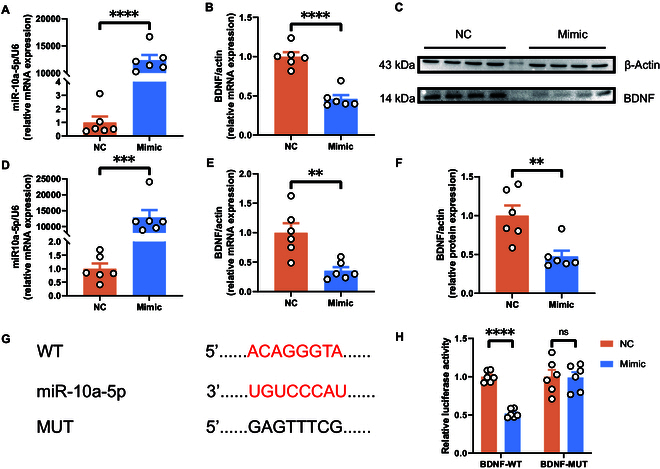
miR-10a-5p directly targets and negatively regulates the expression of BDNF and the identification of binding sites. (A) Transfected mimic-miR-10a-5p into N2A cells to detect the expression of miR-10a-5p. (B) Transfected mimic-miR-10a-5p into N2A cells to detect the expression of BDNF. (C) Protein expression of BDNF from the N2A cells. (D) Transfected mimic-miR-10a-5p into HEK293T cells to detect the expression of miR-10a-5p. (E) Transfected mimic-miR-10a-5p into HEK293T cells to detect the expression of BDNF. (F) Protein expression of BDNF from the N2A cells. (G) Binding site between miR-10a-5p and BDNF. (H) Dual-luciferase assay confirmed the binding site between miR-10a-5p and BDNF. Statistical analysis was performed by Student’s *t* test. *P < 0.05, **P < 0.01, ***P < 0.001, ****P < 0.0001 (*n* = 6).

Exploring the interaction between miR-10a-5p and BDNF, N2A cells transfected with miR-10a-5p mimics exhibited an increase in miR-10a-5p levels alongside a decrease in BDNF expression, confirmed by quantitative reverse transcription PCR (qRT-PCR) and Western blot analyses (Fig. 3A to C and F). This negative regulatory relationship was further validated in human embryonic kidney (HEK) 293T cells, with consistent findings (Fig. 3D and E). The direct targeting of BDNF by miR-10a-5p was confirmed through the construction of wild-type (WT) and mutant plasmids for HEK293T cell transfection (Fig. 3G). Dual-luciferase reporter assays demonstrated a significant reduction in fluorescence intensity in cells transfected with the WT plasmid and miR-10a-5p mimics versus other groups (Fig. 3H), conclusively establishing miR-10a-5p as a direct and negative regulator of BDNF expression.

### Modulation of the miR-10a-5p-BDNF pathway alleviates depressive behaviors in PPD model mice

**Fig. 4.**
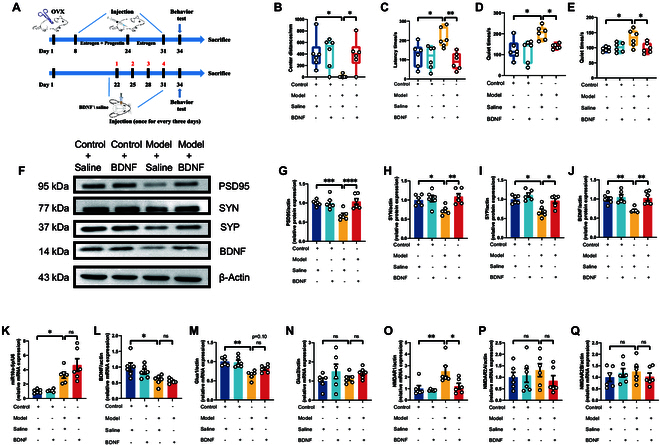
Injecting BDNF protein into the mPFC brain region alleviated the postnatal depression-like phenotype. (A) Modeling and treatment. Behavioral test: (B) Distance the mice traveled in the central region of the open field. (C) Latency time of the mice for their first feeding. (D) Quiet time for mice to undergo forced swimming tests. (E) Quiet time of mice to undergo tail suspension test. Western blot: (F) Protein expression of PSD95, SYN, SYP, and BDNF from the prefrontal cortex. (G) Protein expression of PSD95. (H) Protein expression of SYN. (I) Protein expression of SYP. (J) Protein expression of BDNF. qPCR: (K) mRNA expression of miR-10a-5p. (L) mRNA expression of BDNF. (M) mRNA expression of Glua1. (N) mRNA expression of Glua2. (O) mRNA expression of NMDAR1. (P) mRNA expression of NMDAR2A. (Q) mRNA expression of NMDAR2B. Statistical analysis was performed by 2-way analysis of variance (ANOVA). *P < 0.05, **P < 0.01, ***P < 0.001, ****P < 0.0001 (*n* = 6).

**Fig. 5.**
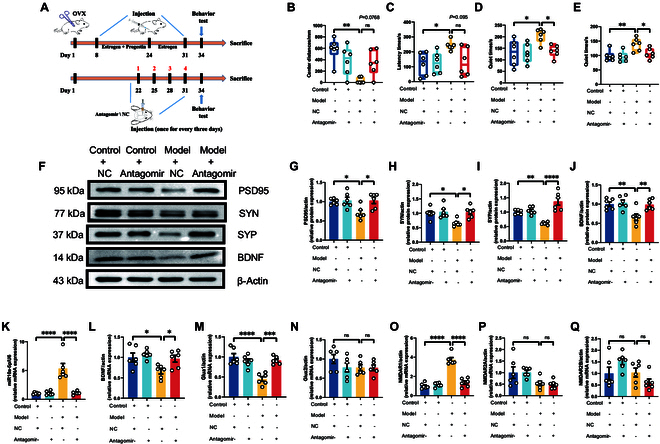
Injecting antagomir of miR-10a-5p into the mPFC brain region alleviated the postnatal depression-like phenotype. (A) Modeling and treatment. Behavioral test: (B) Distance the mice traveled in the central region of the open field. (C) Latency time of the mice for their first feeding. (D) Quiet time for mice to undergo forced swimming tests. (E) Quiet time of mice to undergo tail suspension test. Western blot: (F) Protein expression of PSD95, SYN, SYP, and BDNF from the prefrontal cortex. (G) Protein expression of PSD95. (H) Protein expression of SYN. (I) Protein expression of SYP. (J) Protein expression of BDNF. qPCR: (K) mRNA expression of miR-10a-5p. (L) mRNA expression of BDNF. (M) mRNA expression of Glua1. (N) mRNA expression of Glua2. (O) mRNA expression of NMDAR1. (P) mRNA expression of NMDAR2A. (Q) mRNA expression of NMDAR2B. Statistical analysis was performed by 2-way ANOVA. *P < 0.05, **P < 0.01, ***P < 0.001, ****P < 0.0001 (*n* = 6).

Initiating our investigation with the established animal model detailed in Figs. 4A and 5A, we observed a notable amelioration in depression-like behaviors among the model mice receiving medial prefrontal cortex (mPFC) injections of BDNF or antagomir. These mice demonstrated significantly reduced depressive symptoms across various behavioral assessments, including the open-field, novelty-suppressed feeding, forced swimming, and tail suspension tests (Figs. 4B to E and 5B to E). This improvement underscores the crucial role of BDNF in the pathogenesis of PPD and highlights the therapeutic potential of directly modulating the miR-10a-5p-BDNF pathway in the mPFC region. Furthermore, oxidative stress analyses echoed these positive outcomes, revealing substantial recovery in NO and T-AOC levels in the model groups treated with BDNF or antagomir (Fig. S3). This recovery indicates the efficacy of BDNF and antagomir in counteracting oxidative stress damage characteristic of PPD. Western blot analysis further corroborated these findings, showing a reversal in the diminished expression of synaptic proteins PSD95, SYN, SYP, and BDNF after therapeutic intervention (Figs. 4F to J and 5F to J), thus reinforcing the vital role of these proteins in synaptic health and their contribution to the therapeutic impact of BDNF.

In line with the KEGG analysis outcomes from Fig. 2, which spotlighted the enrichment of miR-10a-5p-related target genes in the glutamate synaptic pathway, our qPCR analyses (Figs. 4K and L and 5K and L) revealed a critical interplay between the dysregulated expression of miR-10a-5p and BDNF, affecting synaptic function. These findings were further validated by assessing the expression levels of key glutamate receptors (Figs. 4M to Q and 5M to Q), establishing a direct link between the modulation of the miR-10a-5p-BDNF pathway and the restoration of glutamatergic signaling in the context of PPD.

### Intranasal delivery of miR-10a-5p antagomir or BDNF as a promising therapeutic approach for PPD

**Fig. 6.**
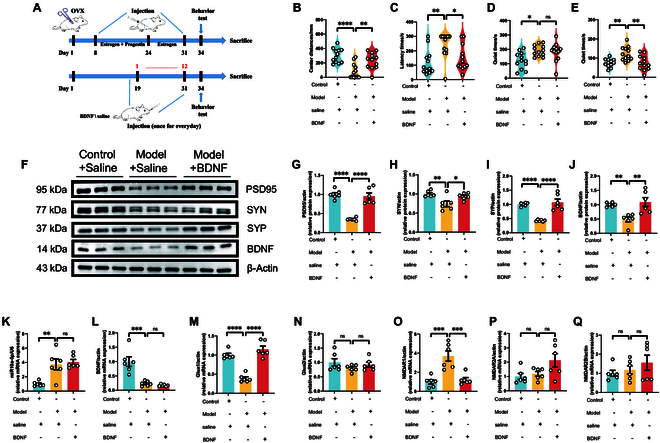
Nasal injection of BDNF protein alleviated the postnatal depression-like phenotype. (A) Modeling and treatment. Behavioral test: (B) Distance the mice traveled in the central region of the open field. (C) Latency time of the mice for their first feeding. (D) Quiet time for mice to undergo forced swimming tests. (E) Quiet time of mice to undergo tail suspension test. Western blot: (F) Protein expression of PSD95, SYN, SYP, and BDNF from the prefrontal cortex. (G) Protein expression of PSD95. (H) Protein expression of SYN. (I) Protein expression of SYP. (J) Protein expression of BDNF. qPCR: (K) mRNA expression of miR-10a-5p. (L) mRNA expression of BDNF. (M) mRNA expression of Glua1. (N) mRNA expression of Glua2. (O) mRNA expression of NMDAR1. (P) mRNA expression of NMDAR2A. (Q) mRNA expression of NMDAR2B. Statistical analysis was performed by 2-way ANOVA. *P < 0.05, **P < 0.01, ***P < 0.001, ****P < 0.0001 (*n* = 12, *n* = 6).

**Fig. 7.**
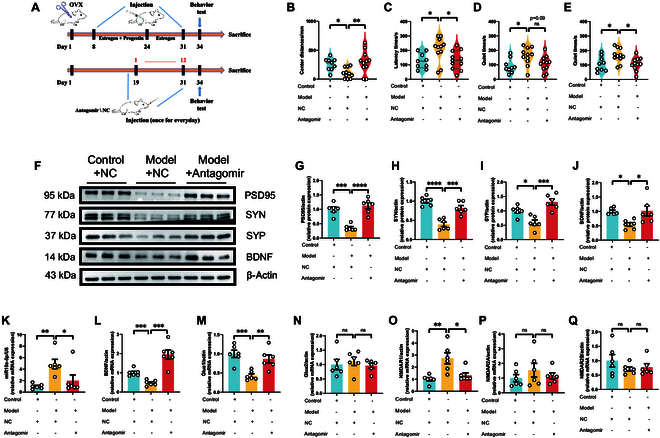
Nasal injection of antagomir alleviated the postnatal depression-like phenotype. (A) Modeling and treatment. Behavioral test: (B) Distance the mice traveled in the central region of the open field. (C) Latency time of the mice for their first feeding. (D) Quiet time for mice to undergo forced swimming tests. (E) Quiet time of mice to undergo tail suspension test. Western blot: (F) Protein expression of PSD95, SYN, SYP, and BDNF from the prefrontal cortex. (G) Protein expression of PSD95. (H) Protein expression of SYN. (I) Protein expression of SYP. (J) Protein expression of BDNF. qPCR: (K) mRNA expression of miR-10a-5p. (L) mRNA expression of BDNF. (M) mRNA expression of Glua1. (N) mRNA expression of Glua2. (O) mRNA expression of NMDAR1. (P) mRNA expression of NMDAR2A. (Q) mRNA expression of NMDAR2B. Statistical analysis was performed by 2-way ANOVA. *P < 0.05, **P < 0.01, ***P < 0.001, ****P < 0.0001 (*n* = 11, *n* = 9, *n* = 6).

Building on the foundational understanding of the miR-10a-5p-BDNF pathway’s role in PPD, we explored the therapeutic potential of noninvasive delivery methods. Intranasal administration of BDNF or miR-10a-5p antagomir offered a novel approach to modulate this pathway, showing significant efficacy in alleviating depressive behaviors in model mice across a spectrum of behavioral tests (Figs. 6A to E and 7A to E). This method’s success highlights its clinical relevance and potential for broader application in treating PPD. Oxidative stress assays further demonstrated the therapeutic value of this approach, with marked improvements in serum CAT, NO, and T-AOC levels following nasal treatment with BDNF or antagomir (Fig. S4). These outcomes not only signify the reversal of oxidative stress damage but also align with the observed behavioral improvements, providing a holistic view of the therapeutic benefits. Moreover, Western blot analyses and qPCR quantification of synaptic and glutamate receptor proteins (Figs. 6F to Q and 7F to Q) confirmed the efficacy of intranasal BDNF or antagomir delivery in restoring normal synaptic function and glutamatergic signaling. Notably, the significant restoration in the expression of glua1 and NMDAR1 following treatment mirrors the effects seen with direct brain injections, underscoring the versatility and effectiveness of nasal administration as a treatment modality.

### The impact of up-regulated miR-10a-5p on synaptic formation in primary neurons

**Fig. 8.**
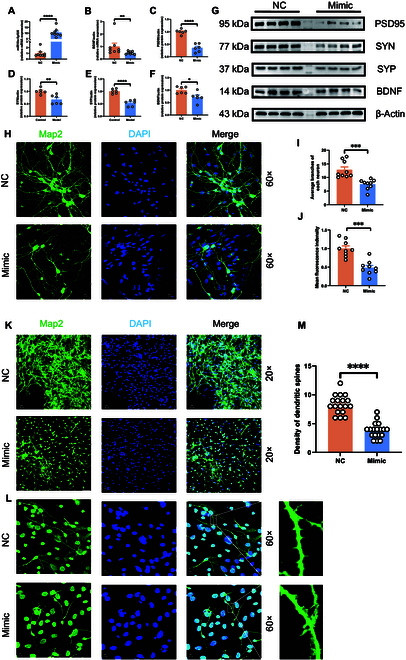
miR-10a-5p transfected into primary neurons affected morphology and gene expression. (A) mRNA expression of miR-10a-5p. (B) mRNA expression of BDNF. (C) Protein expression of PSD95. (D) Protein expression of SYN. (E) Protein expression of SYP. (F) Protein expression of BDNF. (G) Protein expression of PSD95, SYN, SYP, and BDNF from primary neurons. (H) Primary neurons were located using MAP2 and 4′,6-diamidino-2-phenylindole (DAPI) and observed under 60× microscope. Scale bar, 10 μm. (I) Average number of branches per neuron. (J) Fluorescence intensity for each field of view. (K) Primary neurons were located using MAP2 and DAPI and observed under a 20× microscope. Scale bar, 30 μm. (L) Primary neurons were located using MAP2 and DAPI, and dendritic spines were observed under a 60-fold microscope. Scale bar, 10 μm. (M) Density of dendritic spines of primary neurons. Statistical analysis was performed by Student’s *t* test. *P < 0.05, **P < 0.01, ***P < 0.001, ****P < 0.0001 (*n* = 6, *n* = 9, *n* = 18).

Our investigation extended to the cellular level to validate the negative regulatory dynamics between miR-10a-5p and BDNF within primary neurons, specifically focusing on the consequences of miR-10a-5p up-regulation on synaptic formation. Transfecting primary neurons with mimic-miR-10a-5p led to an anticipated increase in miR-10a-5p levels and a corresponding decrease in BDNF expression, as evidenced by qRT-PCR results (Fig. 8A and B). This outcome not only corroborated the miR-10a-5p and BDNF regulatory relationship in a neuronal context but also paralleled findings from our earlier animal model experiments. Further support came from Western blot analyses, which demonstrated diminished expression of synaptic proteins PSD95, SYN, SYP, and BDNF in mimic-transfected neurons (Fig. 8C to G), suggesting a link between PPD and altered protein expression in neurons.

Cellular immunofluorescence studies provided a more nuanced understanding of miR-10a-5p’s role. Neurons transfected with the mimic displayed a notable decrease in synaptic density, branching, and fluorescence intensity relative to the negative control (NC) control group (Fig. 8H to J). It is worth noting that the growth of dendritic spines of neurons transfected with the analog also seemed to be inhibited, and the density of dendritic spines was significantly reduced (Fig. 8K to M). These observations indicate miR-10a-5p’s function as a detrimental regulator of synaptic formation. The overexpression of miR-10a-5p, by negatively regulating BDNF, appears to impede synaptic formation through downstream effects on the expression of critical synapse-associated proteins. This pathway underscores a potential mechanism contributing to the development of PPD via disrupted synaptic architecture.

### The effect of up-regulated miR-10a-5p on the growth and proliferation of primary neural stem cells

Further exploration into miR-10a-5p’s role involved its impact on the proliferation and differentiation of primary neural stem cells (NSCs), achieved through transfecting these cells with mimic-miR-10a-5p. Initial qPCR analyses confirmed the expected modulation of miR-10a-5p and BDNF levels following transfection (Fig. S5A and B). The proliferation of NSCs after transfection was quantitatively assessed using a CCK8 kit, which revealed an inhibitory effect on cell viability in mimic-miR-10a-5p-transfected cells compared to the NC control, indicating that miR-10a-5p overexpression diminishes NSC proliferation capabilities. However, it is noteworthy that cell vitality consistently increased throughout the 7-d observation period (Fig. S5G).

The examination of NSC sphere formation, employing Nestin/SOX2 as NSC markers, further illustrated the negative impact of miR-10a-5p overexpression. Cells transfected with the mimic formed fewer and smaller cell spheres compared to the NC control, with a significant reduction in the total number of spheres and those across various diameters (Fig. S5C to F). These findings suggest miR-10a-5p functions as a negative regulator of NSC proliferation. Interestingly, despite its influence on NSC proliferation, miR-10a-5p does not seem to participate in the differentiation process of these cells. This observation was supported by costaining differentiated NSCs with markers MAP2 and GFAP (Fig. S5H), indicating a distinct regulatory role of miR-10a-5p confined to the proliferation phase of NSC development.

### The role of estrogen receptor 2 in the transcriptional regulation of miR-10a-5p and its impact on neuronal synaptic formation

**Fig. 9.**
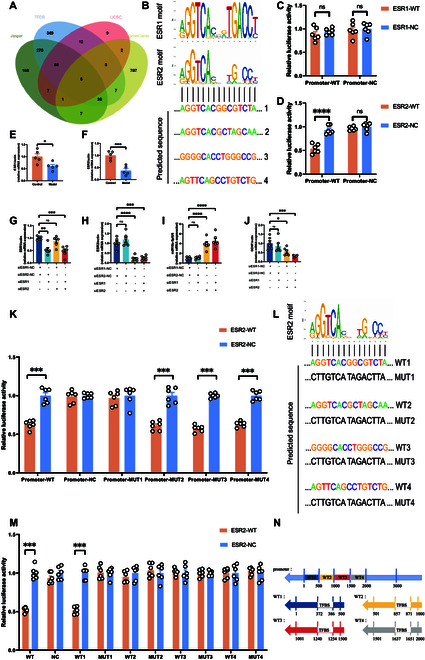
ESR2 acts as a transcription factor to negatively regulate the expression of miR-10a-5p and target to bind its promoter. (A) Venn diagrams screen transcription factors that may bind to the promoter of miR-10a. (B) Possible binding sites between ESR2 and promoter of miR-10a. (C) Dual-luciferase assay showed no binding site between miR-10a promoter and ESR1. (D) Dual-luciferase assay confirmed the binding site between the promoter of miR-10a and ESR2. (E) mRNA expression of ESR1 from the prefrontal cortex. (F) mRNA expression of ESR2 from the prefrontal cortex. siRNA (ESR1) and siRNA (ESR2) were transfected into HEK293T cells: (G) mRNA expression of ESR1. (H) mRNA expression of ESR2. (I) mRNA expression of miR-10a-5p. (J) mRNA expression of BDNF. (K) Dual-luciferase assay confirmed that AGGTCACGGCGTCTA (chr17:48,580,318-48,580,332) was the binding site between miR-10a promoter and ESR2. (L) Possible binding sites and corresponding mutants between ESR2 and miR-10a promoters. (M) Dual-luciferase assay confirmed that AGGTCACGGCGTCTA (chr17:48,580,318-48,580,332) was the binding site between miR-10a promoter and ESR2. (N) The promoter region of miR-10a was divided into four 500-bp fragments, each containing only one predicted binding site. Statistical analysis was performed by Student’s *t* test and 2-way ANOVA. *P < 0.05, **P < 0.01, ***P < 0.001, ****P < 0.0001 (*n* = 6).

Leveraging an array of bioinformatics analyses and transcription factor databases, we embarked on a journey to elucidate the transcriptional intricacies of miR-10a-5p, spanning its primary, precursor, and mature forms. The scarcity of research on pri-miRNAs, particularly their chromosomal identification and sequence procurement, presented a formidable challenge. Addressing this, our team pinpointed the human chromosome region designated for pre-miR-10a, drawing inspiration from the TaqMan Pri-miRNA Assay probe. This led us to earmark an extended locus as the presumptive site for pri-miR-10a, further broadening our search upstream to delineate a putative promoter region. Submission of this sequence to Promoter 2.0 (https://services.healthtech.dtu.dk/services/Promoter-2.0/) facilitated the identification of transcription factors displaying high-affinity binding. Delving deeper, we refined our search to a more stringent 3,000 nucleotide segment upstream (chr17:48,579,947-48,582,946), utilizing AnimalTFDB V4.0 (http://bioinfo.life.hust.edu.cn/AnimalTFDB4/#/TFBS_Predict) and the Jaspar database (https://jaspar.genereg.net), culminating in the prediction of numerous human transcription factors. The culmination of this bioinformatics odyssey, aligned with data from the UCSC database (http://genome.ucsc.edu/), spotlighted 5 transcription factors potentially linked with PPD: ESR2, Nr1h4, Srebf1, Srf, Stat3, and Tbx19 (Fig. 9A).

Amidst fluctuating estrogen levels implicated in PPD, our focus sharpened on ESR2 as a primary transcriptional regulator. Intriguingly, dual-luciferase reporter assays and qPCR analysis on mouse prefrontal cortex tissue revealed diminished expressions of both ESR1 and ESR2 in model mice afflicted with PPD, with ESR2 exhibiting a specific inhibitory interaction with the miR-10a promoter (Fig. 9B to F). To solidify our findings, small interfering RNA (siRNA)-mediated knockdown of ESR1 and ESR2 in HEK293T cells was employed to discern their respective roles in modulating miR-10a-5p and BDNF expressions. The outcomes emphatically demonstrated ESR2’s regulatory capacity over miR-10a transcription and, by extension, BDNF expression, aligning seamlessly with our bioinformatics predictions and experimental results (Fig. 9G to J). Conversely, ESR1’s influence appeared relegated to alternative regulatory pathways, affecting BDNF expression without directly interacting with the miR-10a promoter.

In addition, in order to further confirm the specific binding sites of ESR2 and miR-10a promoters, plasmids containing 4 mutated miR-10a promoters were successively transfected into HEK293T cells and double luciferase report analysis was performed. Finally, AGGTCACGGCGTCTA (chr17:48,580,318-48,580,332) sequence was found to be the specific binding site of ESR2 and miR-10a promoters. As a transcription factor, ESR2 inhibits the activity of miR-10a promoter by binding to this site and ultimately leads to the inhibition of miR-10a transcription (Fig. 9K and L).

To ensure more rigorous analysis and prevent interactions between binding sites, we divided the promoter region of miR-10a into four 500-base pair (bp) fragments, each containing only one predicted binding site. These fragments were then transfected individually into HEK293T cells to assess transcriptional activity. The results confirmed that AGGTCACGGCGTCTA (chr17:48,580,318-48,580,332) remains the sole binding site for the transcription factor ESR2 (Fig. 9M and N), consistent with our previous findings.

### **The impact of diminished ESR2 on synaptic development in primary neuron**s

**Fig. 10.**
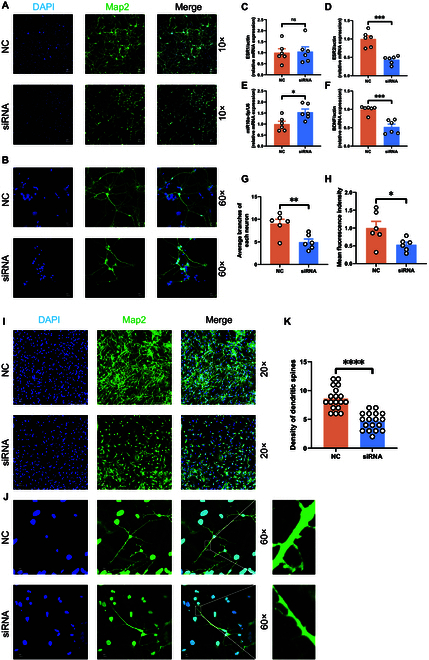
siRNA (ESR2) transfected into primary neurons affected morphology and gene expression. (A) The primary neurons were located using MAP2 and DAPI and observed under 10× microscope. Scale bar, 60 μm. (B) The primary neurons were located using MAP2 and DAPI and observed under 60× microscope. Scale bar, 10 μm. (C) mRNA expression of ESR1. (D) mRNA expression of ESR2. (E) mRNA expression of miR-10a-5p (F) mRNA expression of BDNF (G) Average number of branches per neuron. (H) Fluorescence intensity for each field of view. (I) Primary neurons were located using MAP2 and DAPI and observed under a 20× microscope. Scale bar, 30 μm. (J) Primary neurons were located using MAP2 and DAPI, and dendritic spines were observed under a 60-fold microscope. Scale bar, 10 μm. (K) Density of dendritic spines of primary neurons. Statistical analysis was performed by Student’s *t* test. *P < 0.05, **P < 0.01, ***P < 0.001, ****P < 0.0001 (*n* = 6, *n* = 18).

In a focused investigation to discern the impact of ESR2 on synaptic development, we employed siRNA to selectively down-regulate ESR2 expression in primary neurons. The results of qRT-PCR analysis revealed that reducing ESR2 not only specifically lowered its expression but also led to an increase in miR-10a-5p levels, consequently diminishing BDNF expression. This chain of molecular events corroborates our earlier observations from both bioinformatics analyses and dual-luciferase reporter assays (Fig. 10C to F).

Further exploration using cellular immunofluorescence techniques highlighted a notable decrease in synaptic density, branching, and fluorescence intensity in neurons subjected to ESR2 suppression compared to those treated with a negative control (Fig. 10A, B, G, and H). It is worth noting that the growth of dendritic spines of neurons transfected with the analog also seemed to be inhibited, and the density of dendritic spines was significantly reduced (Fig. 10I to K). These findings underscore the critical influence of ESR2 on synaptic architecture, suggesting that a reduction in ESR2 expression adversely affects synaptic formation in primary neurons.

## Discussion

Our study illuminates the critical dysregulation of the ESR2 gene and its consequential modulation of BDNF gene expression via alterations in miR-10a-5p levels. This intricate molecular interplay leads to a diminished expression of synaptic-related proteins, adversely affecting synaptic plasticity and leading to synaptic damage. Additionally, this cascade of events may extend to affect the expression of glutamate receptor genes, introducing further complexity to the multifaceted pathogenesis of PPD. Compelling evidence from our research indicates that both BDNF and antagomir offer promising therapeutic avenues for mitigating depression-like behaviors in mouse models. Through detailed exploration, we have uncovered the complex molecular mechanisms at play in the onset of PPD, providing essential insights crucial for surmounting the existing hurdles in diagnosis and treatment strategies for this condition. Our findings not only shed light on the biological underpinnings of PPD but also highlight potential targets for therapeutic intervention, offering hope for more effective management and treatment options for affected individuals.

Currently, miRNAs are heralded as pivotal elements in the precise diagnosis and targeted treatment of diseases, including a spectrum of psychiatric disorders like depression [29–35]. Nevertheless, the role of miRNAs in PPD has not been as extensively explored, marking an area ripe for discovery and understanding of underlying pathological mechanisms. Our approach to bridge this gap involved leveraging bioinformatics to identify miRNAs associated with PPD and verifying their relevance through qPCR technology. The selection process underscored the significant role of the prefrontal cortex in emotional regulation, leading to the identification of miR-10a-5p as a key regulator of the BDNF gene, potentially playing a crucial role in the etiology of PPD. This finding not only enriches our understanding but also directs future research toward unraveling the complexities of this condition. Further substantiating the importance of miR-10a-5p, our study demonstrates that targeted intervention using antagomir, an inhibitor of miR-10a-5p, significantly ameliorates symptoms of PPD, as evidenced by both behavioral and molecular improvements. This highlights the potential of miR-10a-5p as an essential target in the therapeutic landscape of PPD. Our findings align with existing research that illustrates the neuroprotective role of miR-10a-5p regulation. For instance, a study on chronic pain and depression showed that a regimen of short-term stress treatment led to the down-regulation of miR-10a-5p in the prefrontal cortex, suggesting its involvement in enhancing neuroprotection and possibly contributing to its antidepressant effects [36]. Moreover, the connection between miR-10a-5p and stress, a prevalent factor in both PPD and the general onset of depression, underscores the intricate relationship between miR-10a-5p regulation and mental health [37]. Intriguingly, research indicating miR-10a-5p’s involvement in the pathogenesis of autism by modulating Grm3 expression further supports its role as a critical neuroprotective agent in mental health disorders [38]. Additionally, miR-10a-5p’s regulatory impact extends to inflammation, a known correlate of PPD, thereby positioning miR-10a-5p as a vital link between neuroinflammation and PPD [39].

Given the pivotal role of miRNA in regulating gene expression through epigenetic mechanisms across various psychiatric disorders, there’s a pressing need to delve deeper into the downstream targets of miR-10a-5p, particularly brain-derived neurotrophic factor (BDNF), within the context of PPD. This exploration is crucial for unraveling the molecular intricacies behind PPD, focusing on the miRNA–mRNA regulation mode. Our animal studies have revealed significant differences in BDNF expression levels in the prefrontal cortex between model and control mice, underscoring a direct link between BDNF’s dysregulation and the onset of PPD. This observation aligns with the prevailing hypothesis that BDNF is a key risk factor for PPD, supported by evidence of its association with depression under adverse conditions [16,22,40–45]. BDNF’s critical functions in enhancing synaptic plasticity, fostering neurogenesis, and ensuring neuronal survival highlight its potential as a candidate gene for PPD. Literature reviews further corroborate the relationship between abnormal BDNF expression or gene polymorphisms and PPD, suggesting that BDNF may influence the development of this condition. Despite the recognized connection between BDNF and PPD, there has been a lack of detailed research on the precise mechanisms through which BDNF contributes to the disorder. To address this gap, our study extended the investigation to synapse-related proteins such as SYP, SYN, and PSD95 in both model and control mice. Our findings demonstrate a correlation between the reduced expression of these proteins and PPD, reinforcing the hypothesis that synaptic dysfunction plays a significant role in the disease’s pathogenesis. This conclusion is supported by existing research, including studies on the effects of treatments like Timosaponin B-III, selective serotonin reuptake inhibitors (SSRIs), and progesterone therapy on PPD models, which have highlighted abnormalities in BDNF and synapse-related protein expression [46–48]. In pursuit of a deeper understanding, we conducted cell transfection experiments to manipulate BDNF expression in primary neurons, revealing that reduced BDNF levels detrimentally affect neuronal branching and synaptic development. This suggests that BDNF’s influence on synaptic plasticity could be a critical factor in the emergence of PPD. Leveraging this insight, we explored therapeutic interventions targeting BDNF, administering the protein directly to the prefrontal cortex and nasally. This approach successfully mitigated depression-like behaviors, illustrating BDNF’s therapeutic potential for PPD. Our research not only sheds light on the molecular pathways implicated in PPD but also offers promising directions for future therapeutic strategies, emphasizing the importance of BDNF and related synaptic proteins in combating this complex condition.

While existing literature underscores the importance of miRNA–mRNA interactions in the development of PPD, there remains a notable gap in understanding the origins of miRNAs and their relationship to hormonal fluctuations, particularly estrogen, which is recognized as a primary risk factor for this condition. Despite acknowledging hormonal changes as triggers for PPD, the detailed pathways through which these fluctuations exert their influence remain largely unexplored. The dramatic shifts in estrogen levels from pregnancy to postpartum are thought to increase vulnerability to environmental stressors, positioning these hormonal changes as key contributors to the onset of perinatal depression. Consequently, the role of genetic and epigenetic mechanisms related to estrogen and its receptors has come under scrutiny as potential underpinnings of perinatal depression. Some research has linked estrogen receptor genes with PPD scores, suggesting that alterations in serotonergic or dopaminergic pathways might play a role in the disorder [2,18,49–52]. Yet, the exact pathophysiological mechanisms and how gene polymorphisms may contribute to PPD are still not fully understood.

A review posited that the roots of PPD lie in genetic predispositions to major depression, exacerbated by environmental influences, particularly the modulatory effects of estrogen fluctuations through estrogen signaling pathways [53]. This perspective advocates for considering ESR1 and ESR2 genes in postnatal depression research. Our study focuses specifically on the postpartum aspect of the condition, examining the interactions between estrogen receptor genes and PPD. Given the predominant roles of ESR1 and ESR2, and the existing ambiguity regarding their specific contributions to the onset of PPD, we investigated both receptors separately. Our animal experiments highlighted distinct expression patterns of ESR1 and ESR2 in the prefrontal cortex between model and control mice. Through bioinformatics analysis and dual-luciferase assays, we identified ESR2’s critical function in neuronal synaptic plasticity, where it acts as a transcription factor influencing miR-10a expression, thereby modulating BDNF levels and affecting synaptic plasticity. Cell transfection experiments lent further support to these insights, showing that targeting ESR2 with siRNA significantly altered miR-10a and BDNF levels, whereas ESR1-targeted siRNA did not produce comparable effects. This highlights ESR2’s essential role in synaptic health. Immunofluorescence studies reinforced these findings, showing that reducing ESR2 expression impairs normal neuronal development.

**Fig. 11.**
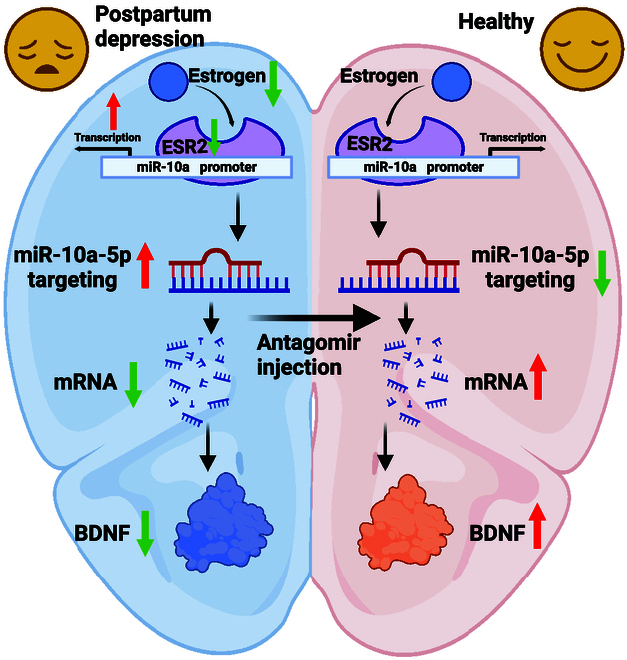
Estrogen-dependent estrogen receptor 2 with reduced expression in the prefrontal cortex can act as a transcription factor to negatively regulate the transcription of pri-miR-10a, thus leading to an increase in the content of miR-10a-5p, which further promotes the negative regulatory process of miR-10a-5p on BDNF, resulting in a decrease in the expression of BDNF. This in turn affects neuroprotection and promotes postnatal depression-like behavior. However, this process can be treated by Antagomir, which indirectly restores the expression level of BDNF by reducing the content of miR-10a-5p, enhancing neuroprotective ability and curing PPD-like behavior.

Current research on PPD often focuses on the analysis of individual depression-related genes, which limits the ability to comprehensively assess the impact of complex molecular interactions on the disorder. Our study advances this field by exploring the interplay between estrogen receptors, miRNA, BDNF, and other genes in relation to PPD. Significantly, we have established the molecular biological relationship between estrogen, ESR2, and miRNA. Interestingly, parallels can be drawn between our findings and certain studies on breast cancer. For instance, research by Bozkurt et al. [54]. in 2020 indicated that cells lacking the estrogen receptor exhibited higher expressions of miR-10a-5p compared to those with active estrogen receptors, suggesting a potential inverse relationship between the estrogen receptor and miR-10a-5p. Moreover, the link between estrogen and miR-10a-5p was separately highlighted in studies by Zhang et al. [55] and Gao et al. [56] in 2019 and 2023, respectively, providing partial validation of the molecular connection between estrogen and miR-10a-5p. However, these studies stopped short of delving into the direct interaction among estrogen, estrogen receptor β, and miRNA. Our research fills this critical gap by demonstrating that estrogen-dependent estrogen receptor β in the prefrontal cortex can negatively regulate the transcription of pri-miR-10a. This regulation establishes a crucial link between miR-10a-5p and BDNF, thereby influencing neuroprotection and contributing to PPD-like behaviors (Fig. 11).

A significant limitation in current research is the insufficient differentiation between PPD and general depression, which hinders follow-up studies and leaves a critical gap in understanding the unique characteristics of PPD. The defining feature of PPD lies in its specific postpartum context, making it essential for research to focus on this period for more accurate conclusions. While identifying depression-related genes like BDNF has been a useful strategy, we argue that research should also explore genes linked to the physiological changes occurring before, during, and after pregnancy to better capture the unique female-specific aspects of PPD. This approach should not dismiss the overlap between PPD and general depression but rather investigate how genes related to physiological changes, such as ESR2, interact with those associated with depression to uncover PPD’s underlying pathological mechanisms. Our study aims to elucidate the molecular regulatory networks between these potential risk genes, advancing the clinical identification of biomarkers and refining diagnostic criteria for PPD. In doing so, we hope to enhance the understanding of both the similarities and distinctions between PPD and general depression, ultimately leading to improved therapeutic strategies.

In conclusion, our research sheds light on the complex pathogenesis of PPD and introduces promising avenues for therapeutic intervention. Our findings demonstrate that depressive behaviors can be effectively mitigated through targeted injections of antagomir-miR-10a-5p or BDNF directly into the prefrontal cortex. More significantly, we propose intranasal administration of BDNF or antagomir-miR-10a-5p as an innovative and practical treatment strategy for PPD, combining efficacy with ease of application. The consistency of our experimental outcomes with established understandings of PPD etiology not only validates our approach but also opens new pathways for the identification of biomarkers and the advancement of precision medicine in this domain. We look forward to further investigations that build upon our work, deepening our molecular and cellular insights into PPD’s true nature and potentially revolutionizing its management and treatment.

## Materials and Methods

### Animals and the establishment of PPD model

Female balc/c mice were purchased from the Peking University Health Science Center (Beijing, China). They were all sexually mature virgins of approximately the same weight and 12 weeks of age. All animals were housed in standard rooms with a humidity of 50 ± 1% and a temperature of 24 ± 1 °C. Day and night were divided according to a 07:00 light/21:00 dark schedule, during which time all animals were provided with adequate amounts of food and water. Animal surgery was approved by the Animal Care and Use Committee of Minzu University, China. Considering the modeling methods of many studies, we established a model of PPD according to the following conditions: The mice were anesthetized with isoflurane gas, the back of the mice was disinfected with alcohol before surgery, and both ovaries were removed from the back. For the PPD model group, 0.1 ml of vegetable oil containing estradiol (0.5 μg/d) and progesterone (0.8 mg/d) was injected intraperitoneally into the mice 7 d after recovery from ovary resection for 16 d, and then 0.1 ml of vegetable oil containing estradiol (10 μg/d) was injected intraperitoneally into the mice for 7 d to establish the PPD model group. For the sham surgery control group, the ovaries were removed without incision, and 7 d after recovery from sham surgery, 0.1 ml of vegetable oil was continuously treated for 23 d to eliminate possible errors caused by surgical wounds.

### Behavioral tests

To detect depression-like behavior in mice, after establishing the PPD mouse model, we conducted the open-field, novelty-suppressed feeding, forced swimming, and tail suspension tests. In the open-field test, we mainly measured the movement distance of mice in the open-field box. In the novelty-suppressed feeding test, we measured the incubation period for animals to approach and chew food in a new environment. It should be noted that mice should be allowed to fast for 24 h before the test. In the forced swimming test, we paid more attention to the resting time when the mice entered a state of despair. In the tail suspension test, the time the mice gave up struggling and stopped exercising was the focus of our analysis. All behavioral experiments were conducted 3 to 6 d after the modeling procedure, with only one behavioral test performed per day. The experiments were conducted in the following order: open-field test, novelty-suppressed feeding test, forced swimming test, and tail suspension test.

### Oxidative stress marker level/activity measurement

Following the behavioral tests, blood samples were collected from the mice, and serum was isolated to evaluate oxidative stress status. Several key markers were measured, including SOD, CAT, T-AOC, NO, GSH, and MDA. All assays were conducted using kits from Nanjing Jiancheng Bioengineering Institute (Nanjing, China), and the experimental procedures followed the manufacturer’s official guidelines [57].

### Bioinformatics analysis

Genes strongly associated with PPD were screened through the Gene Cards, Disgenet, and Diseases databases, and the key genes for PPD research were selected by choosing the intersection of the results retrieved from these 3 databases. Furthermore, PPI network analysis was conducted for these key genes through the String database. The PPI network result was passed through Cytoscape software, and the Betweenness value of each gene and interaction was calculated for sequencing and graph output by the built-in app CytoNCA. Subsequently, the online website (https://www.bioinformatics.com.cn/) was used to perform GO analysis, focusing on the interaction between genes and KEGG enrichment analysis. For miRNAs, they were screened through the Gene Cards database and confirmed in animal brain tissues using qPCR technology. The target genes of the miRNAs under study were predicted through 4 databases: TargetScan, miRDB, miRPath, and miRWalk, and the key genes were selected by identifying the overlapping target genes. The subsequent steps of PPI network analysis and GO and KEGG enrichment analysis remained consistent with the content described above [58]. It is important to note that “postpartum depression” should be used as the search term when screening for risk genes associated with PPD, while “miR-10a-5p” should be used as the search term when screening for target genes regulated by miR-10a-5p.

### Western blot

Protein extraction: Brain tissue samples were treated with radioimmunoprecipitation assay lysate on ice, followed by centrifugation to extract the proteins. The reagents for protein extraction were procured from Beyotime Biotechnology (Beijing, China).

Protein quantification and denaturation: The concentration of extracted proteins was determined using the BCA protein assay kit from Beyotime Biotechnology. For heat-induced denaturation, we utilized sodium dodecyl sulfate–polyacrylamide gel electrophoresis loading buffer from the same supplier.

Western blotting procedure: Primary antibodies for actin, SYP, SYN, and PSD95 were purchased from Cell Signaling Technology (Boston, USA), while the antibody for BDNF was obtained from Abcam (Cambridge, UK).

### RNA extraction and qRT-PCR

Total RNA was extracted from the hippocampus and prefrontal cortex tissues of mice using Trizol reagent (Thermo Fisher Scientific, Waltham, MA, USA) to extract total RNA. We used the RNA reverse transcription kit of Gene Star (Beijing, China) and followed the experimental procedures recommended by the manufacturer to convert mRNA into cDNA. After the primers were constructed, real-time fluorescent quantitative PCR was performed using Gene Star’s 2*RealStar Green Fast Mixture kit to analyze the predicted miRNA levels and normalized them to the expression of U6. All qRT-PCRs were performed using the LightCycler 96 real-time PCR assay system (Roche, Basel, Switzerland).

Primer sequences:

U6-F: ACGCTTCACGAATTTGCGTGTC

U6-R: CTCGCTTCGGCAGCACATATACT

miR-146a-5p-F: GGGTGAGAACTGAATTCCA

miR-146a-5p-R: CAGTGCGTGTCGTGGAGT

miR-212-5p-F: GGGACCTTGGCTCTAGACTG

miR-212-5p-R: CAGTGCGTGTCGTGGAGT

miR-212-3p-F: GGGTAACAGTCTCCAGTCA

miR-212-3p-R: CAGTGCGTGTCGTGGAGT

miR-210-5p-F: GGGAGCCACUGCCCACCGC

miR-210-5p-R: CAGTGCGTGTCGTGGAGT

miR-10a-5p-F: GGGTACCCTGTAGATCCGAA

miR-10a-5p-R: CAGTGCGTGTCGTGGAGT

miR-21-5p-F: GGGTAGCTTATCAGACTGA

miR-21-5p-R: CAGTGCGTGTCGTGGAGT

miR-126-3p-F: GGGTCGTACCGTGAGTAAT

miR-126-3p-R: CAGTGCGTGTCGTGGAGT

miR-125b-5p-F: GGGTCCCTGAGACCCTAAC

miR-125b-5p-R: CAGTGCGTGTCGTGGAGT

actin-F: AGATCAAGATCATTGCTCCTCCT

actin-R: CTCAGTAACAGTCCGCCTAGAA

BDNF-F: GCCTCCTCTACTCTTTCTGC

BDNF-R: ATGGGATTACACTTGGTCTC

Glua1-F: GACTGTGAATCAGAACGCCTCAAC

Glua1-R: GTCACATTGGCTCCGCTCTC

Glua2-F: TCCACTTCGGAGTTCAGACT

Glua2-R: AAAACTGGGAGCAGAAAGCA

NMDAR1-F: ACCCCAAGATCGTCAACATCG

NMDAR1-R: TATCTTCCAAGAGCCGTGTCG

NMDAR2A-F: GGTCAGCTTGAAAACTGGGAAG

NMDAR2A-R: GTGGTGGCAAAGATGTACCC

NMDAR2B-F: TGCTGCTCATTGTCTCTGCT

NMDAR2B-R: CTTTGCCGATGGTGAAAGAT

### Cell transfection

Mimic-miR-10a-5p/mimic-NC and siRNA/NC were ordered from IBSBIO (Shanghai, China) and transfected into N2A or HEK293T cell. The specific experimental procedure was as follows:

The cell culture medium (Dulbecco’s modified Eagle’s medium high-glucose medium containing 10% fetal bovine serum and 1% antibiotics) was configured, the cell concentration was adjusted to 10^5^ cells/ml, and the cells were planted in 24-well cell culture plates. It is worth noting that the reagents and media we use for cell culture are sourced from HyClone (Logan, Utah, USA). About 24 h after cell growth, the cells were replaced with a new serum-free and antibiotic-free cell culture medium. Cell transfection began when the cell density reached about 70% after 3 h of continuous culture.

The Lipo3000 kit from Invitrogen (Carlsbad, CA, USA) is a liposome transport method with a good transfection effect, so the cell miRNA transfection experiment is performed according to the Lipo3000 kit.

### Plasmid construction, extraction, and sequencing verification

Identifying ACAGGGTA as the possible binding site of miR-10a-5p and BDNF gene from the TargetScan database, we purchased 2 kinds of *Escherichia coli* DH5α containing BDNF WT plasmid and BDNF mutant plasmid, respectively, from Weizhenbio Company (Wuhan, China). A total of 471 bp of the 3′ untranslated region (UTR) sequence of the BDNF gene (including the core sequence ACAGGGTA) was constructed onto the pmirGLO dual-luciferase reporter vector and named the BDNF WT plasmid. A total of 471 bp of the 3′UTR mutant sequence of the BDNF gene (including the core sequence GAGTTTCG) was constructed into the pmirGLO dual-luciferase reporter vector and named the BDNF mutant plasmid. In addition, a 3,000-bp sequence (human chromosome chr17:48,579,947-48,582,946) is built to the upstream of F-Luciferase as a pGL3-Promoter promoter, and the pGL3-Basic empty promoter is designed in the same way. The pCMV-MCS-flag system inserted ESR2 sequences as overexpressed ESR2 transcription factor plasmids, and similarly designed empty transcription factor vectors. Similarly, we also identified 4 possible binding sites for the promoter of ESR2 and miR-10a (chr17,48,580,318-48,580,332: AGGTCACGGCGTCTA; chr17,48,580,803-48,580,817: AGGTCACGCTAGCAA; chr17,48,581,186-48,581,200: GGGGCACCTGGGCCG; chr17,48,581,583-48,581,597: AGTTCAGCCTGTCTG) and, on this basis, mutated successively into CTTGTCATAGACTTA and constructed 4 mutant constitution particles.

The 3,000-bp upstream promoter region of miR-10a was divided into four 500-bp fragments. The 1- to 500-bp fragment was designated as WT1, with a core sequence at positions 372 to 386 bp (AGGTCACGGCGTCTA). The 501- to 1,000-bp fragment was named WT2, with a core sequence at positions 857 to 871 bp (AGGTCACGCTAGCAA). The 1,001- to 1,500-bp fragment was designated as WT3, with its core sequence at positions 1,240 to 1,254 bp (GGGGCACCTGGGCCG). Lastly, the 1,501- to 2,000-bp fragment was named WT4, with a core sequence at positions 1,637 to 1,651 bp (AGTTCAGCCTGTCTG). Each fragment was carefully designed to contain only one predicted binding site, and WT and mutant plasmids were constructed accordingly for further analysis.

The kit used to extract the plasmid from *E. coli* was from TIANGEN Company (Beijing, China), and the service of sequencing the extracted plasmid was provided by Sangon Biotech (Beijing, China) with CMV-F and SV40pA-R primers. All the experimental steps were carried out in accordance with the manufacturer’s recommended methods.

### Dual-luciferase analysis

HEK293T cells, renowned for their robust transfection capability, were chosen as the preferred cell line for our subsequent experiments. The transfection protocol employed here mirrors that applied to N2A cells, with the distinction that it begins by transfecting either the BDNF WT or mutant plasmids, followed by subsequent transfections of mimic-miR-10a-5p or mimic-NC at 3-h intervals. The dual-luciferase reporting kit utilized in this study was generously provided by Promega (Madison, WI, USA), and the procedure was meticulously executed in strict accordance with the comprehensive protocol supplied by the company. In a similar fashion, the transcriptional regulation of pri-miR-10a by the ESR2 and ESR1 gene, serving as a transcription factor, was validated using identical methodologies.

### Brain stereotaxic injection

By confirming the negative regulation of BDNF by miR-10a-5p, we further verified the pathogenesis of depression-like behavior mediated by PPD. In addition, aiming to explore the therapeutic effect of antagomir-miR-10a-5p and BDNF on PPD model mice, we ordered the antagonist antagomir-miR-10a-5p and the antagonist control antagomir-Nc from IBSBIO (Shanghai, China). The human recombinant BDNF mature protein was purchased from Novoprotein (Shanghai, China). The solution concentration of the antagonist antagomir-miR-10a-5p and antagomir-NC was adjusted to 100 μmol/ml, and the solution concentration of BDNF protein was adjusted to 1 μg/ml. Whether it is antagomir-miR-10a-5p, antagomir-NC, BDNF, or normal saline, we do brain stereotaxic injections. The ovariectomy was used as the first day of modeling, and the 22nd day was used as the first day of stereotaxic injection therapy. One microliter was injected into each side of the mPFC brain region on the left and right sides every 3 d. Finally, 3 d after the stereotactic brain injection was completed, we then performed behavioral tests on the mice. The stereotaxic coordinates for mPFC injection were anterior posterior (AP) +1.75 mm, medial lateral (ML) ±0.25 mm, and dorsal ventral (DV) −1.90 mm.

### Nasal treatment

The solution concentrations of the antagonists antagomir-miR-10a-5p and antagomir-NC were adjusted to 100 μM, and the solution concentrations of BDNF protein were adjusted to 50 μg/ml. Whether it was antagomir-miR-10a-5p, antagomir-NC, BDNF, or saline, we used bilateral nasal administration with 10-μl injection per day on each side. The ovariectomy was used as the first day of modeling, and the 19th day was used as the first day of nasal treatment, which was performed once a day for a total of 12 times. Waiting for 3 d after the final nasal treatment was completed, we then performed behavioral tests on the mice [59].

### Extraction, culture, transfection of primary neurons, and morphological observation

Conventionally, primary neurons are derived from rat embryos at 18 d of gestation. To investigate the impact of miR-10a-5p on neuronal synaptogenesis, we isolated single cells from neural spheres obtained from embryonic brain tissue. These cells were then plated in 24-well dishes. After 4 d of culture, we adjusted the final concentration of mimic-miR-10a-5p or mimic-NC to 50 nM following the recommended protocol from Abbos and transfected them into the neuronal cells using the Lipo3000 transfection kit. The same method was also applied to investigate the effects of siRNA-ESR2 or siRNA-NC on primary neuron cells.

Ninety-six hours after transfection, we examined synaptic branch number and fluorescence intensity using MAP2 antibodies from Cell Signaling Technology in Boston, USA, and a Leica DMi8 microscope in Solms, Germany. In addition, we also observed the growth of dendritic spines of primary neurons 14 d after transfection, which were also treated with MAP2 antibody. We captured images and processed the relevant data using ImageJ software.

For each treatment group, 9 distinct cell slide samples were used to assess fluorescence intensity and synaptic branching. Additionally, 18 different cell slide samples were employed per group to examine dendritic spines in neurons. Each cell slide contained at least 3 separate microscope fields for analysis. In each 60× magnification field, a minimum of 6 to 8 primary neurons were observed, and the data were averaged across these observations to ensure statistical accuracy.

### Extraction, culture, transfection, proliferation, and differentiation of primary NSCs and morphological observation

By convention, the primary NSCs were taken from the embryos of rats at 13.5 d of gestation.

To explore the effect of miR-10a-5p on the proliferation ability of NSCs, neural spheres of embryonic brain tissue were digested into single cells and inoculated into 96-well plates. After 2 d of culture, NSCs into spheres were obtained as neural sphere clones. The final concentration of mimic-miR-10a-5p or mimic-NC was adjusted to 50 nM according to the recommended protocol of Abbos and transfected into NSCs according to the reference procedure of the Lipo3000 transfection kit. Cell viability was measured by Beyotime’s CCK8 kit (Beijing, China) at 24, 48, 72, 96, 120, and 144 h after transfection, and cell proliferation curves were plotted.

For the neurosphere assay, the number and size of NSCs grown into neural spheres are observed by Leica DMi8 (Leica, Solms, Germany) after transfection for 72 h, images are taken, and the relevant data processing is performed by ImageJ software.

### Statistical analyses

All data analyses were performed using GraphPad Prism 9.0 software, with results expressed as the mean ± SEM (standard error of the mean). Multivariate data were analyzed using 2-way analysis of variance (ANOVA), followed by Tukey’s post hoc test for pairwise comparisons. For comparisons between 2 groups, statistical significance was assessed using Student’s *t* test. A *P* value of less than 0.05 was considered statistically significant.

## Data Availability

All data included in this study are available upon request by contacting the corresponding author.
