# Supplementary material for: Deciphering and Targeting the ESR2–miR-10a-5p–BDNF Axis in the Prefrontal Cortex: Advancing Postpartum Depression Understanding and Therapeutics
Source: Research (Wash D C). 2024 Nov 25;7:0537. doi: 10.34133/research.0537 (PMC11586475; doi:10.34133/research.0537)

Supplemet Figure 1. Bioinformatics analysis of genes associated with postpartum depression. (A) Vennchart screens for genes associated with postpartum depression. (B) Interaction between genes associated with postpartum depression. KEGG terms: (C) KEGG analysis of genes associated with postpartum depression. GO terms: (D) Analysis of biological processes of genes related to postpartum depression. (E) Analysis of cellular component of genes related to postpartum depression. (F) Analysis of molecular function of genes related to postpartum depression.

Supplement Figure 2. Bioinformatics analysis of target genes of miR-10a-5p. (A) KEGG analysis of target genes of miR-10a-5p. (B) KEGG analysis of target genes of miR-10a-5p. (C) Distribution of target genes in Estrogen signaling pathway. (D) Distribution of target genes in glutaminergic synapse.

Supplement Figure 3. After injecting BDNF protein into mPFC brain region, the serum oxidative stress level of mice was detected: (A) The CAT content in serum of mice. (B) The NO content in serum of mice. (C) The T-AOC content in serum of mice. (D) The GSH content in serum of mice. (E) The MDA content in serum of mice. (F) The SOD content in serum of mice. After injecting antagomir into mPFC brain region, the serum oxidative stress level of mice was detected: (G) The CAT content in serum of mice. (H) The NO content in serum of mice. (I) The T-AOC content in serum of mice. (J) The GSH content in serum of mice. (K) The MDA content in serum of mice. (L) The SOD content in serum of mice. Statistical analysis was performed by two-way analysis of variance (ANOVA). ∗, $p < 0.05$, ∗∗, $p < 0.01$, ∗∗∗, $p < 0.001$ (n=6).

Supplement Figure 4. The level of serum oxidative stress in mice was detected after nasal administration of BDNF protein: (A) The CAT content in serum of mice. (B) The NO content in serum of mice. (C) The T-AOC content in serum of mice. (D) The GSH content in serum of mice. (E) The MDA content in serum of mice. (F) The SOD content in serum of mice. The level of serum oxidative stress in mice was detected after nasal administration of antagomir: (G) The CAT content in serum of mice. (H) The NO content in serum of mice. (I) The T-AOC content in serum of mice. (J) The GSH content in serum of mice. (K) The MDA content in serum of mice. (L) The SOD content in serum of mice. Statistical analysis was performed by two-way analysis of variance (ANOVA). ∗, $p < 0.05$, ∗∗, $p < 0.01$, ∗∗∗, $p < 0.001$ (n=7).

Supplemet Figure 5. miR-10a-5p affected the morphology, proliferation and gene expression of neural stem after transfection of primary neural stem cells. (A) The mRNA expression of miR-10a-5p (n=8). (B) The mRNA expression of BDNF (n=8). (C) Total number of primary neural stem cells (n=5). (D) The number of primary neural stem cells with multiple diameters (0 to 50μm, 50 to 100μm, and greater than 100μm). (E) The primary neural stem cells were located using Nestin, SOX2 and DAPI and observed under 20x microscope. Scale bar, 30μm. (F) The primary neural stem cells were located using Nestin, SOX2 and DAPI and observed under 60x microscope. Scale bar, 10μm. (G) Changes in proliferative ability of primary neural stem cells at 0-144 hours (n=6). (H) MAP2, GFAP and DAPI were used to locate neurons and glial cells derived from primary neural stem cells and observed under a 10x microscope. Scale bar, 60μm.

Statistical analysis was performed by Student’s t-test. ∗ , $p < 0.05$, ∗ ∗ , $p < 0.01$, ∗ ∗ ∗ , $p < 0.001$, ∗ ∗ ∗ ∗ , $p < 0.0001$.

Supplement Figure 1

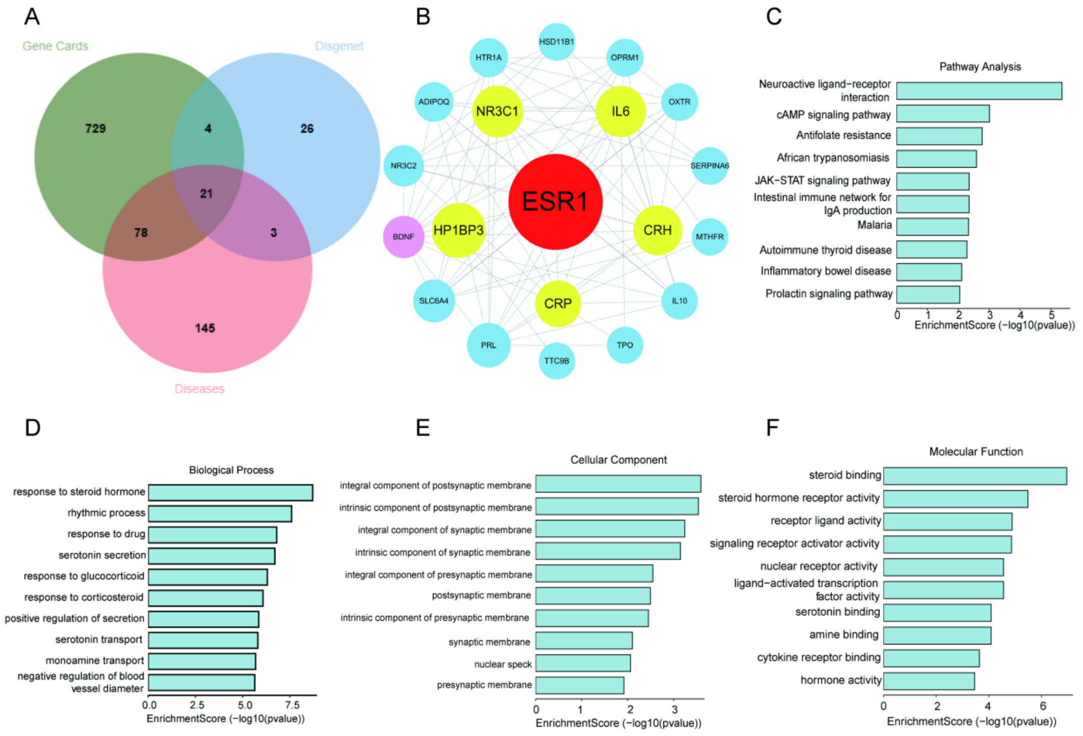

Supplement Figure 2

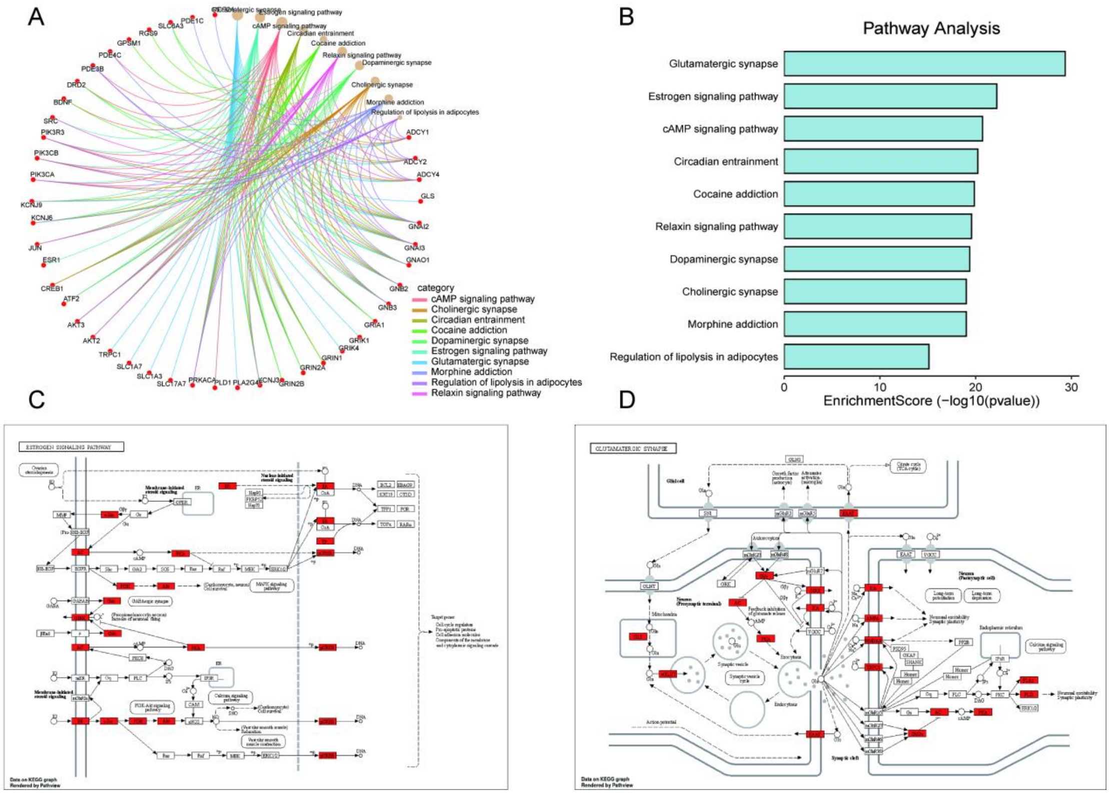

Supplement Figure 3

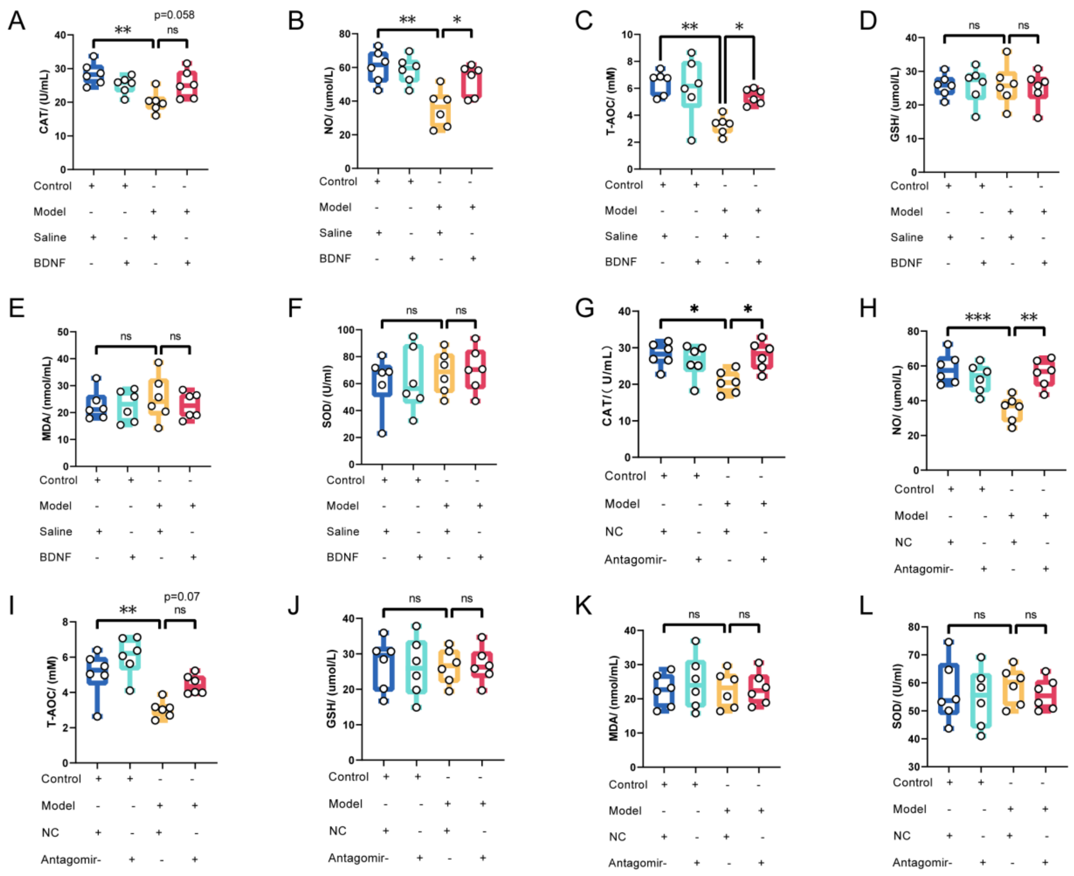

Supplement Figure 4

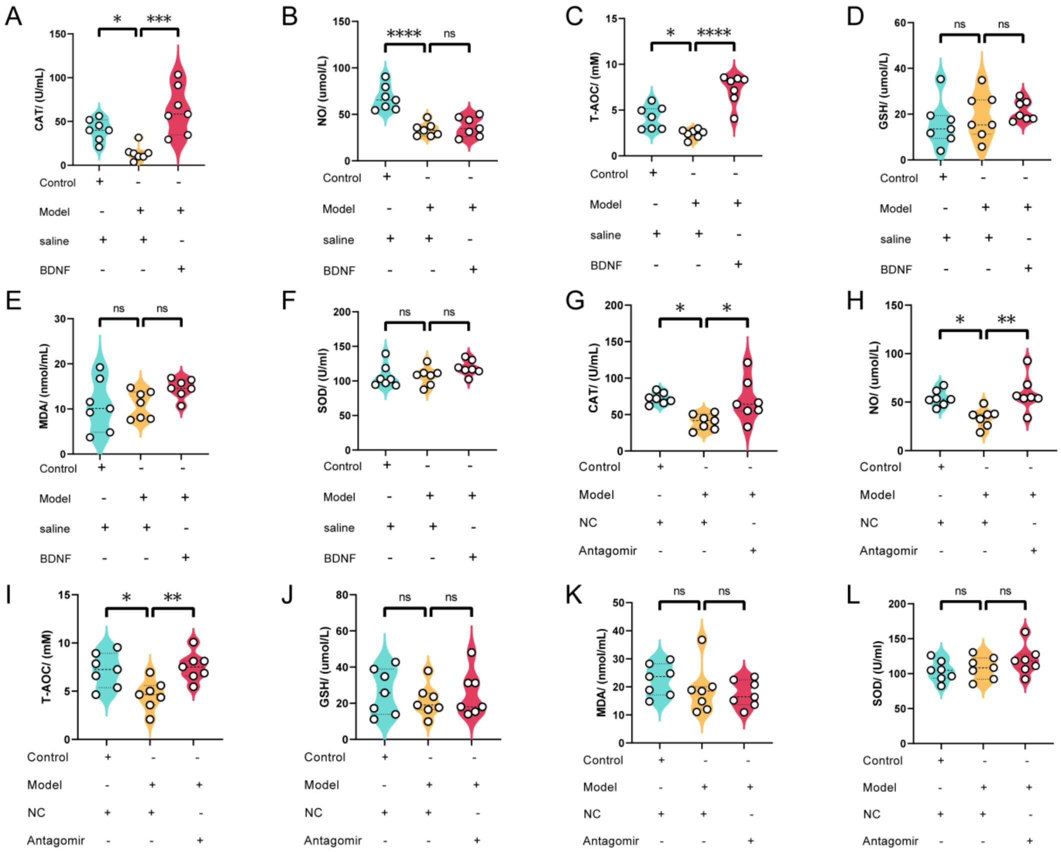

Supplement Figure 5

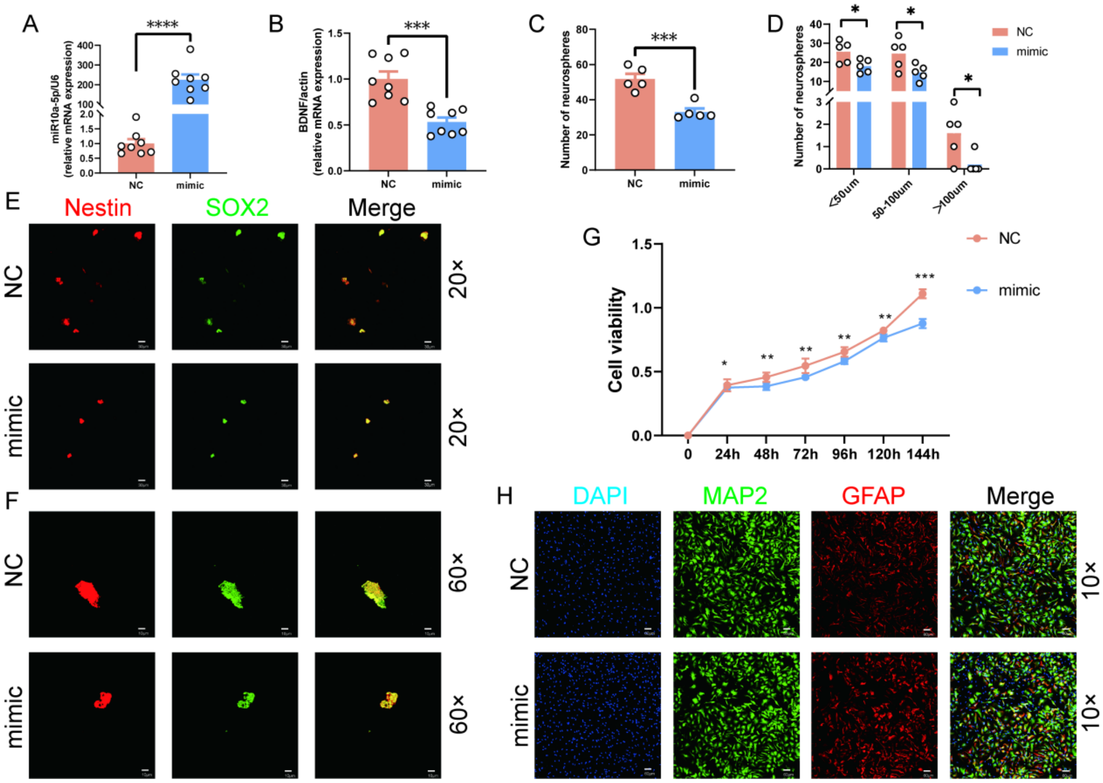

Supplement: Supplementary 1 — Figs. S1 to S5 [file research.0537.f1.zip › Supplementary Materials.pdf]
