# Supplementary figures and images for: Deciphering and Targeting the ESR2–miR-10a-5p–BDNF Axis in the Prefrontal Cortex: Advancing Postpartum Depression Understanding and Therapeutics
Source: Research (Wash D C). 2024 Nov 25;7:0537. doi: 10.34133/research.0537 (PMC11586475; doi:10.34133/research.0537)

A

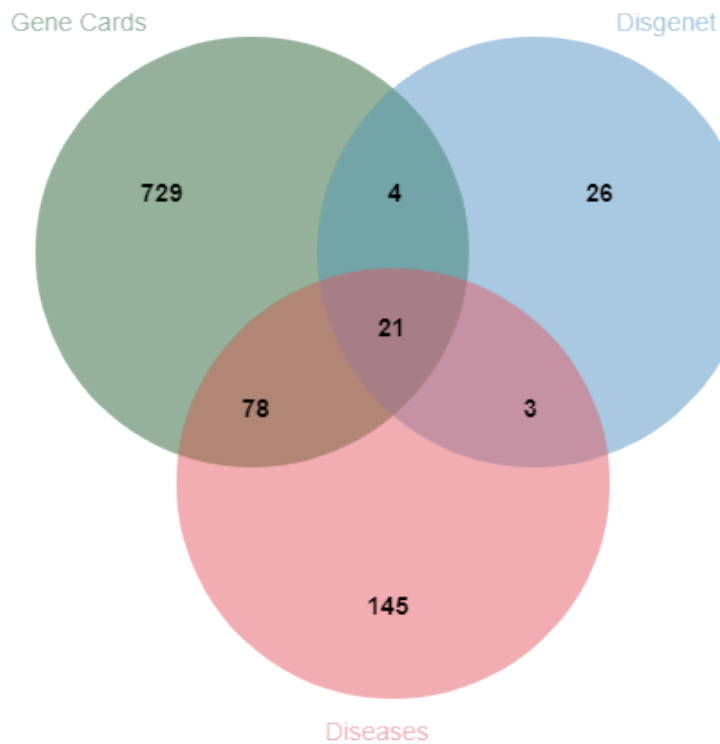

B

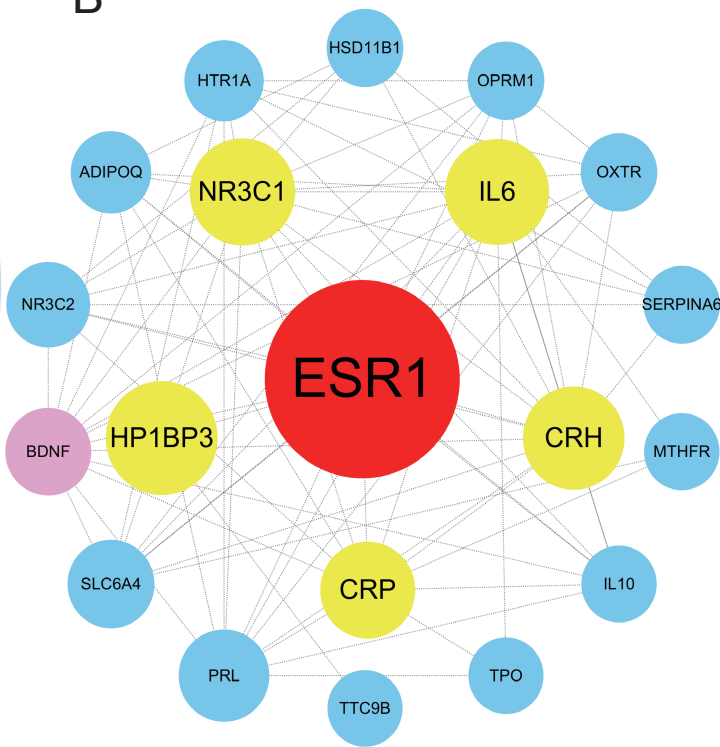

C

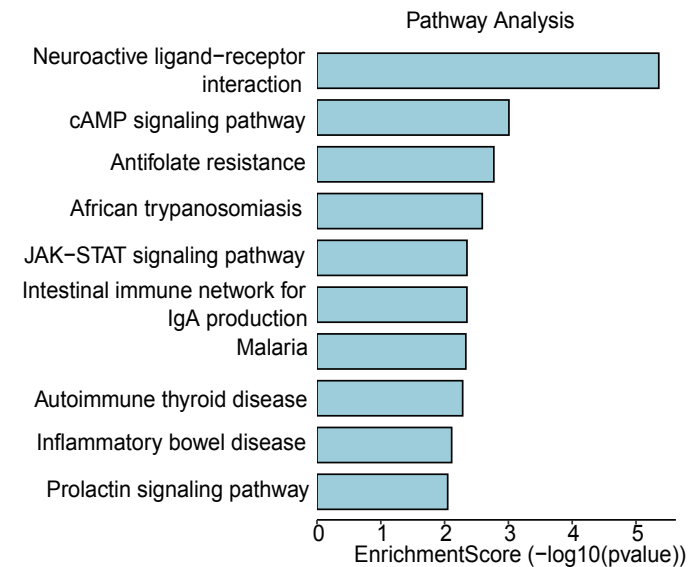

D

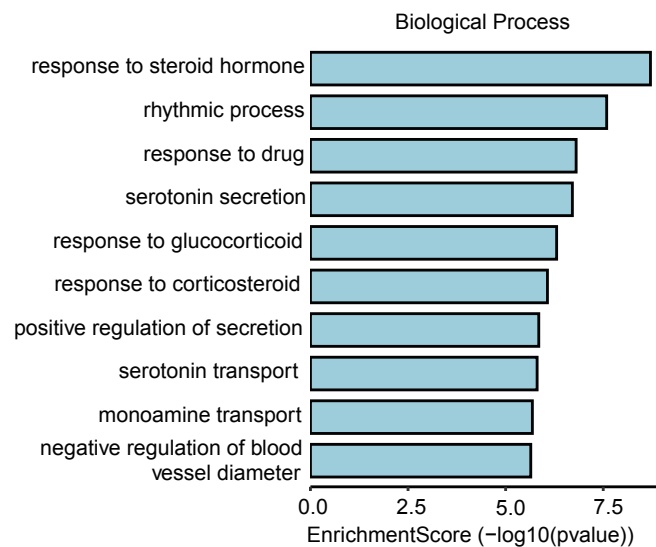

E

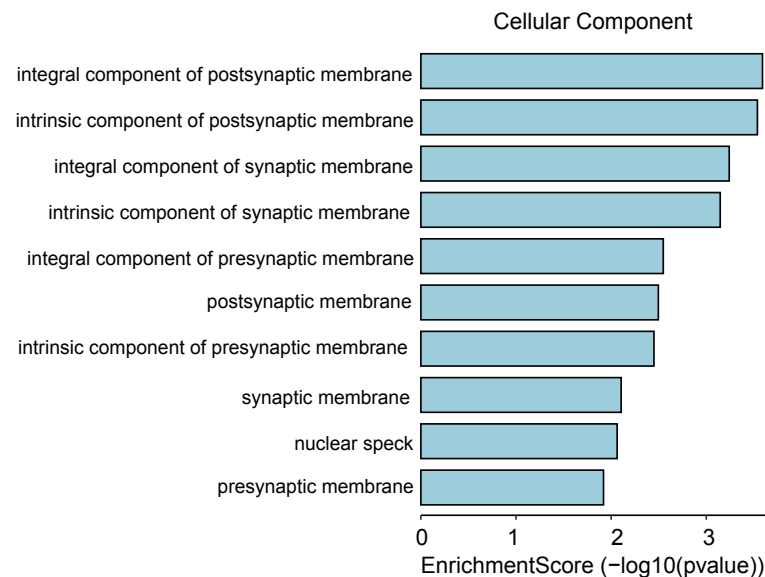

F

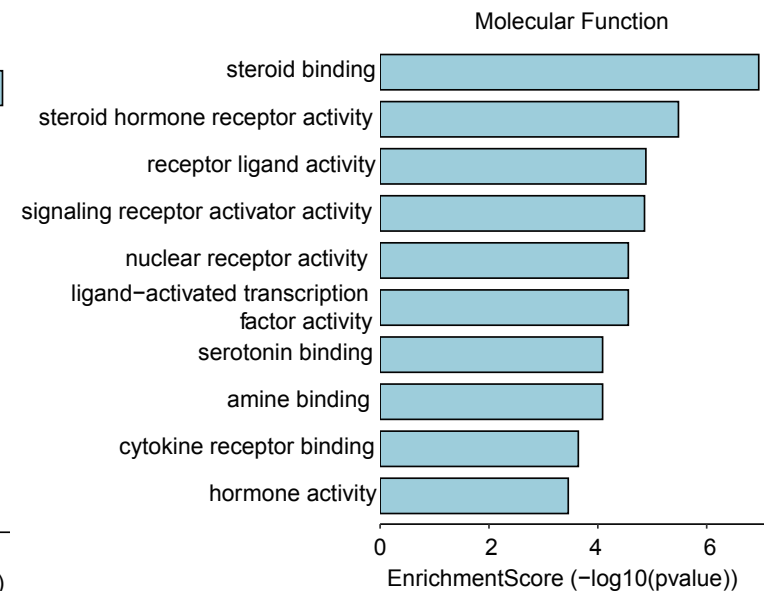

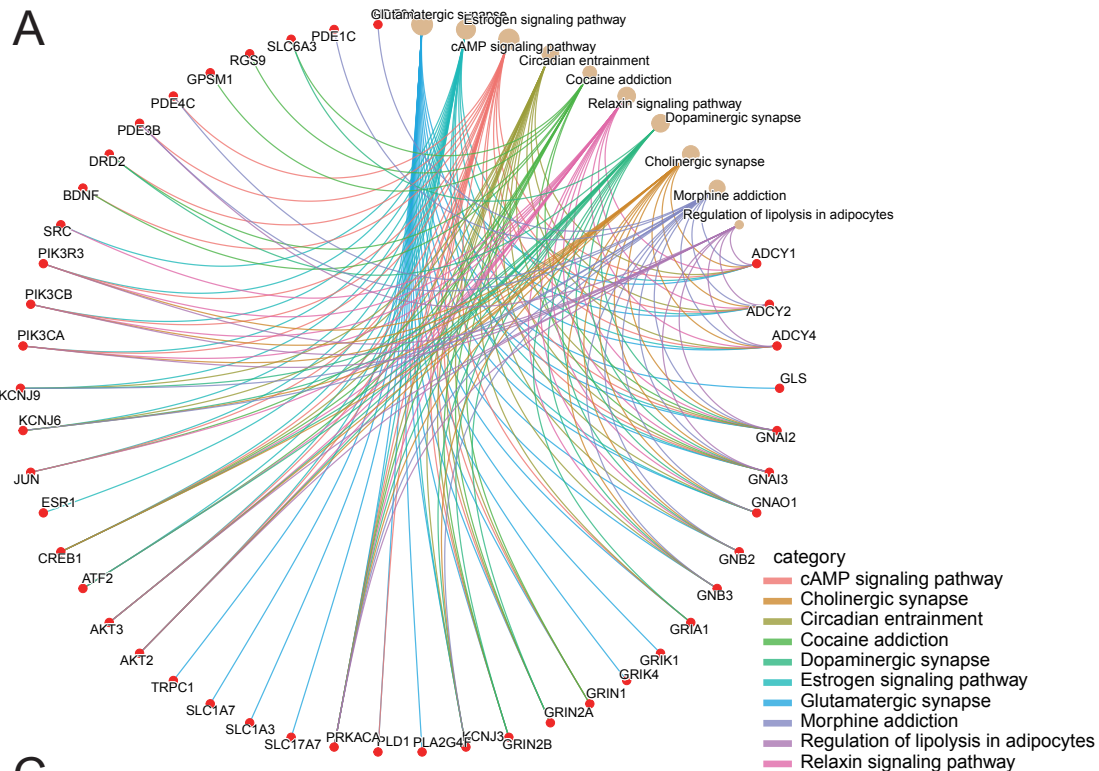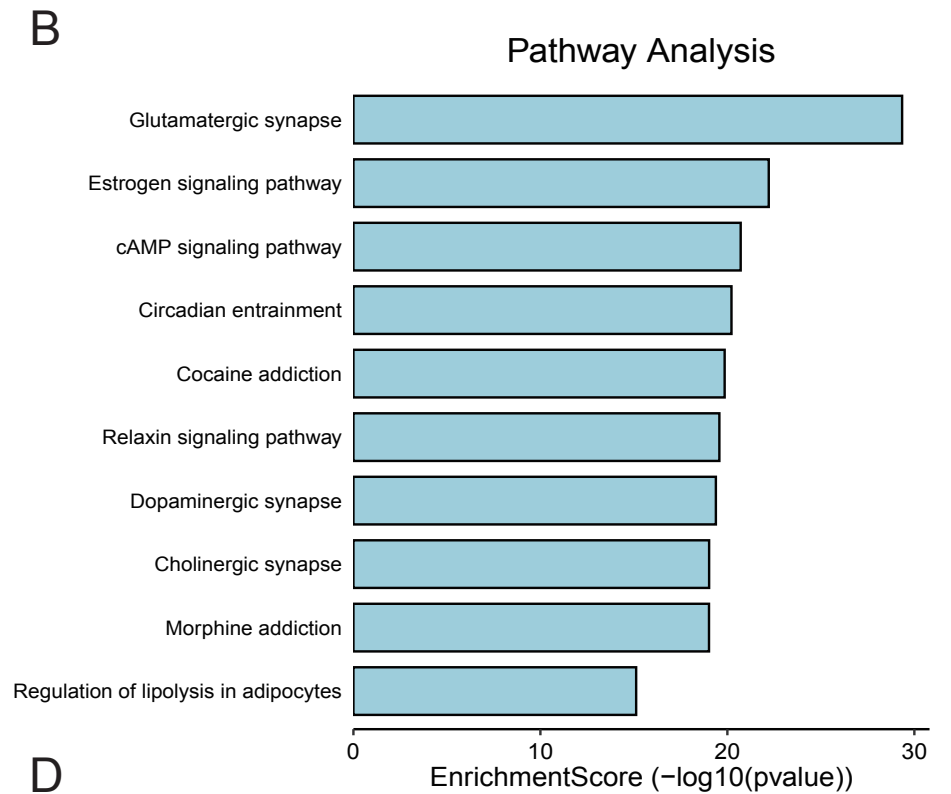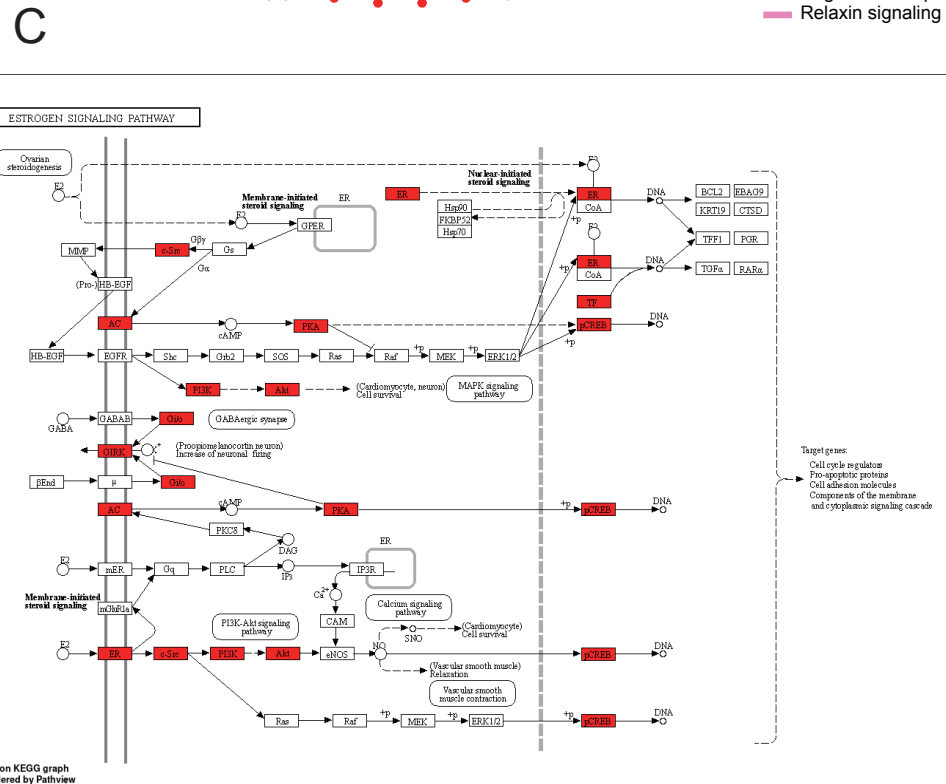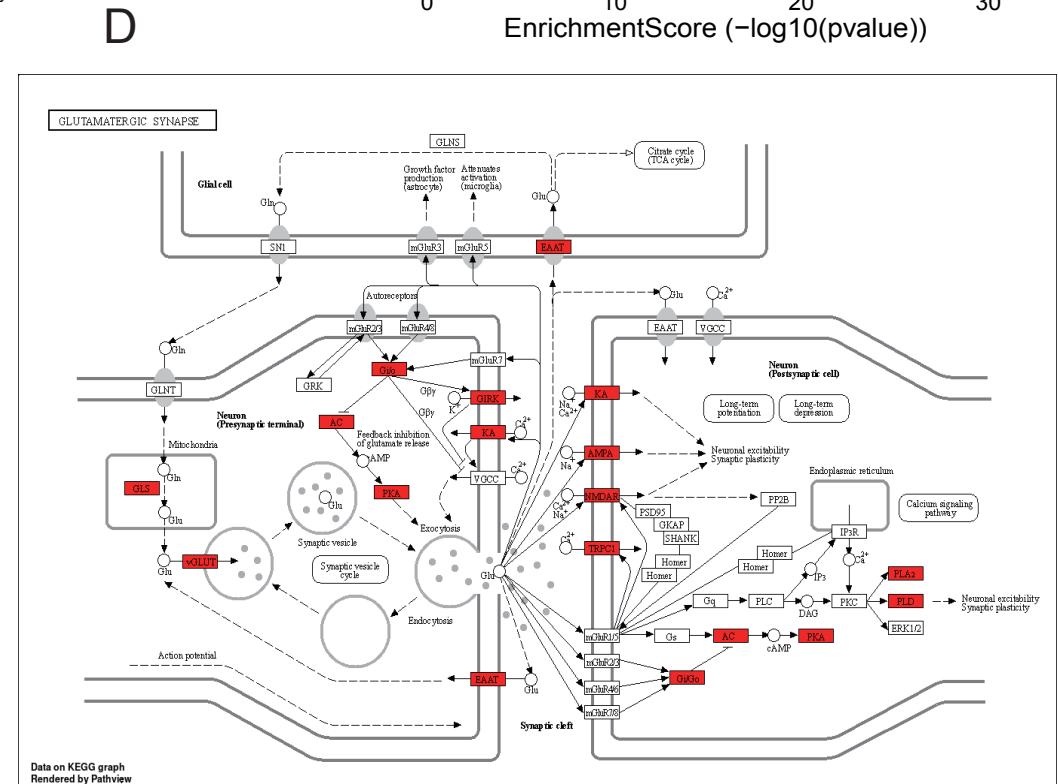

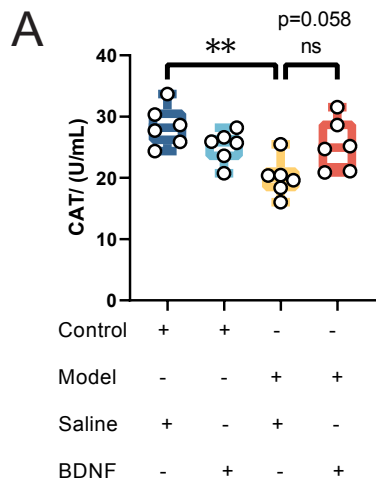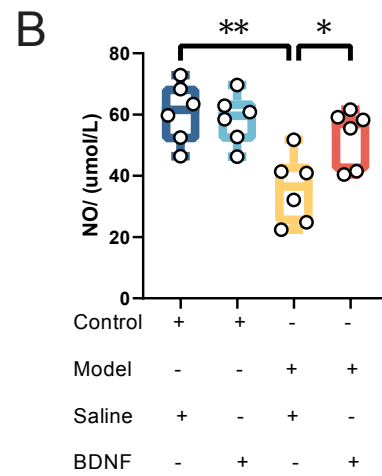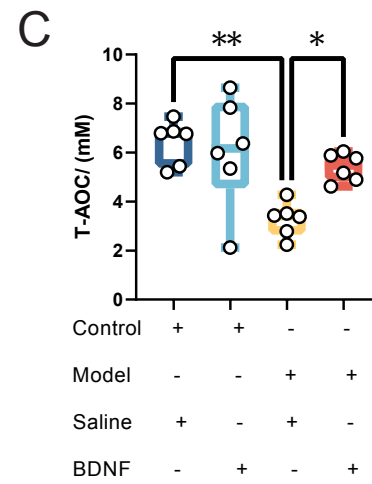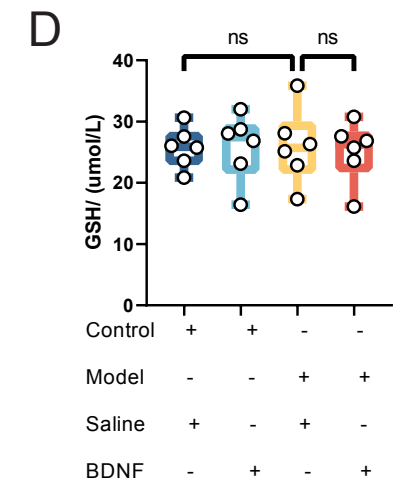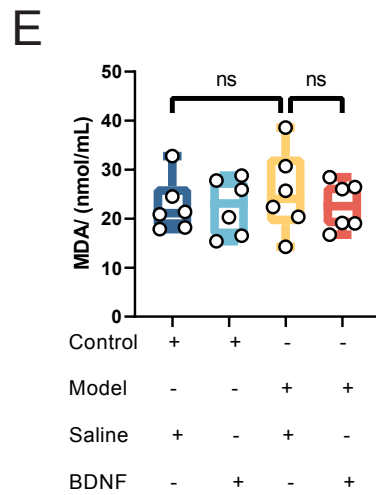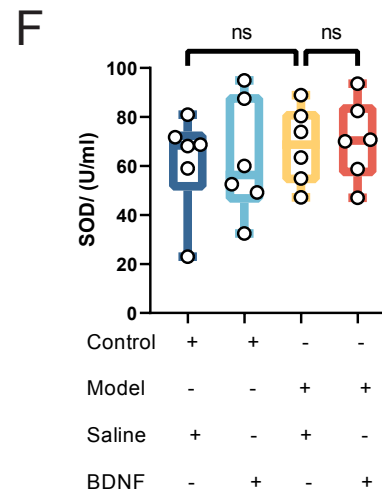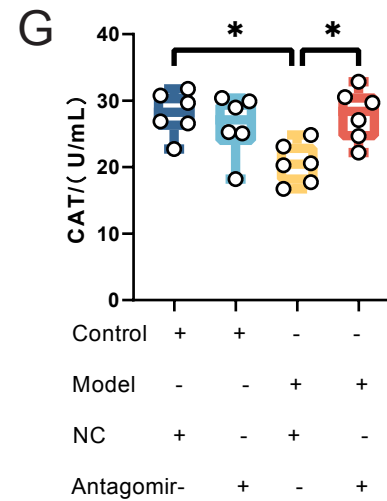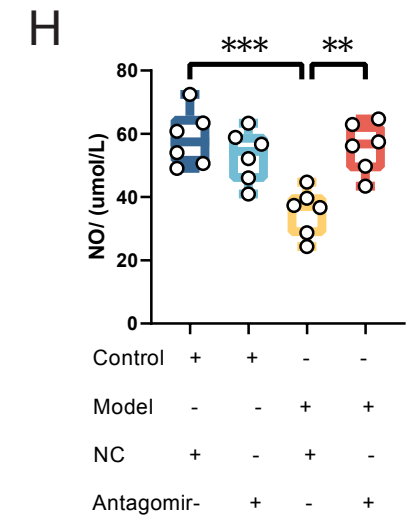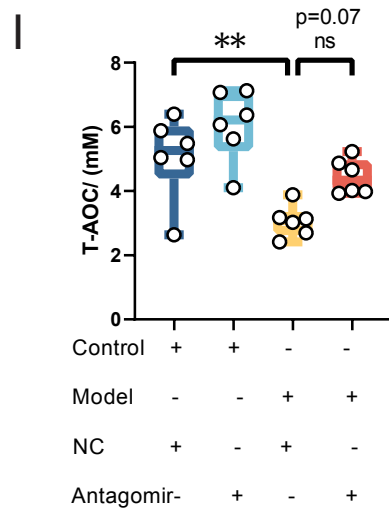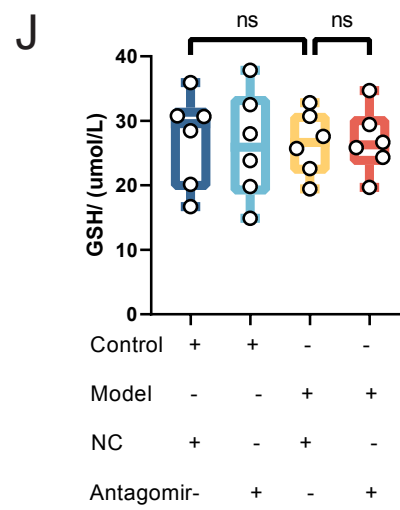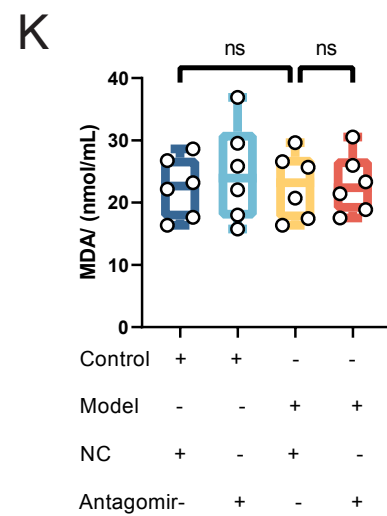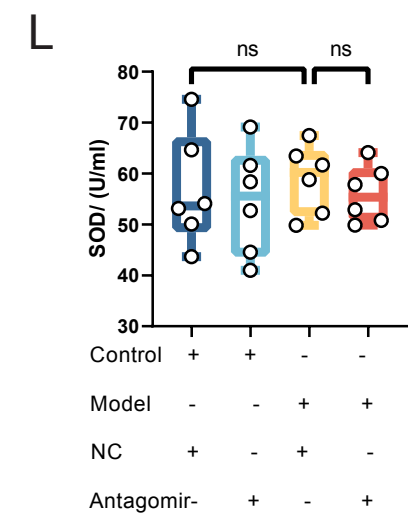

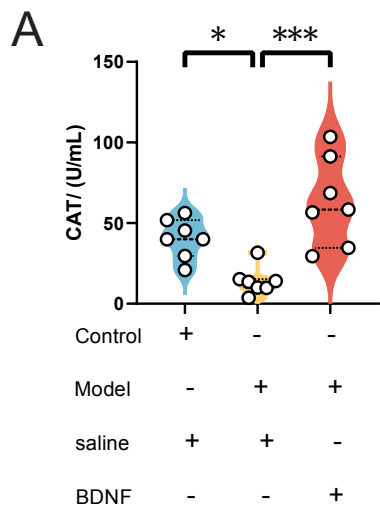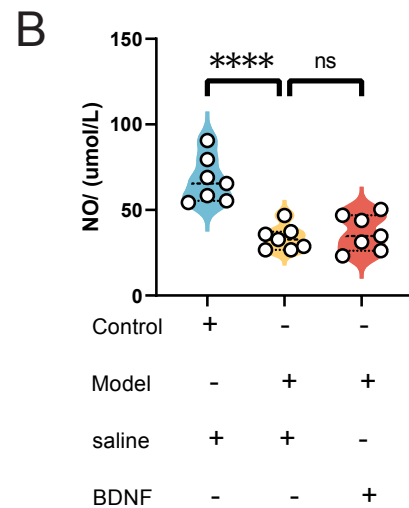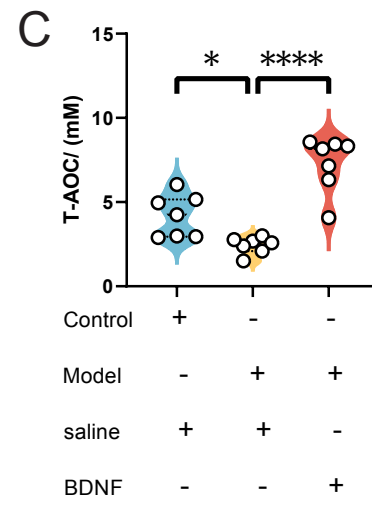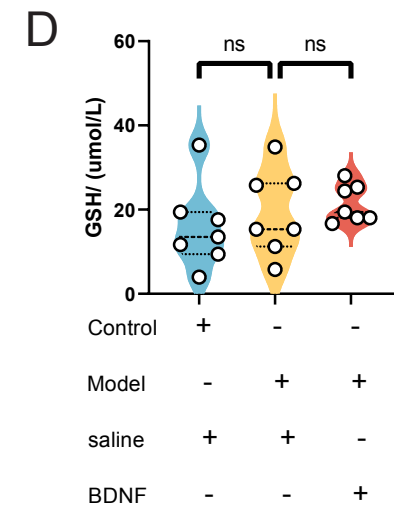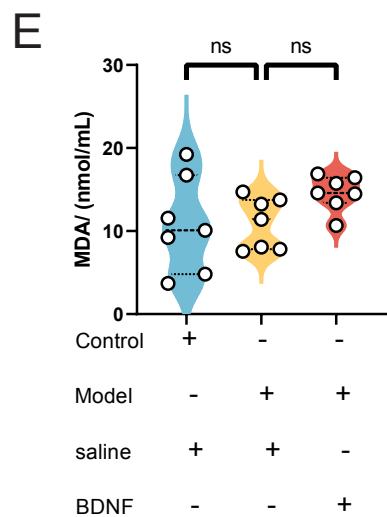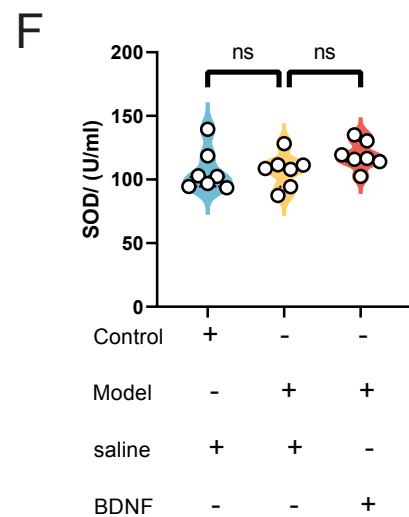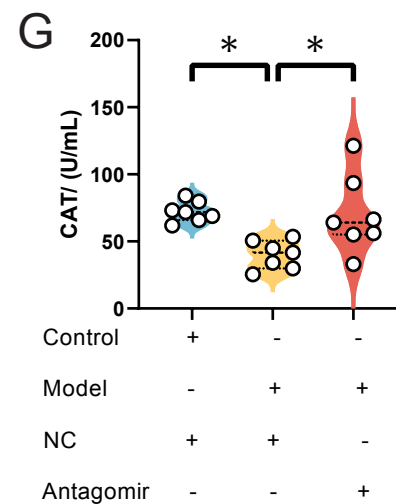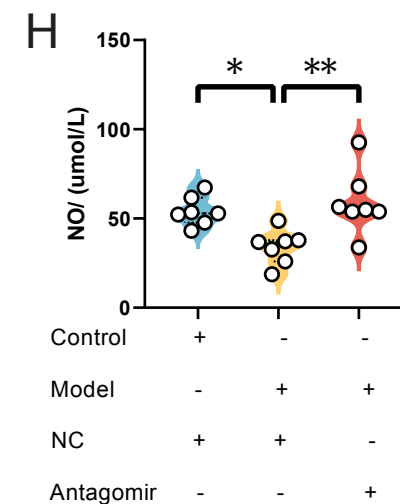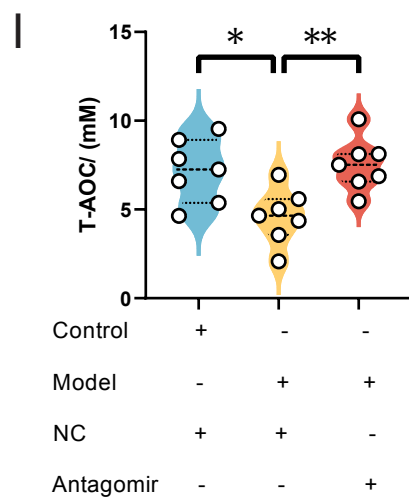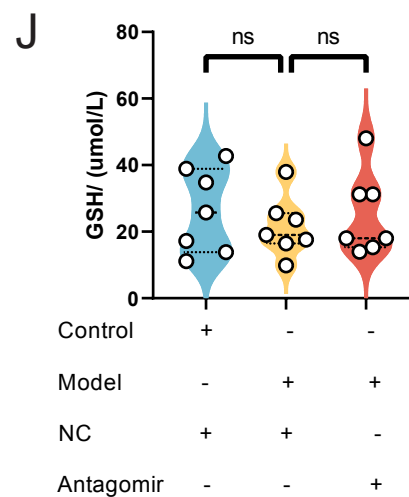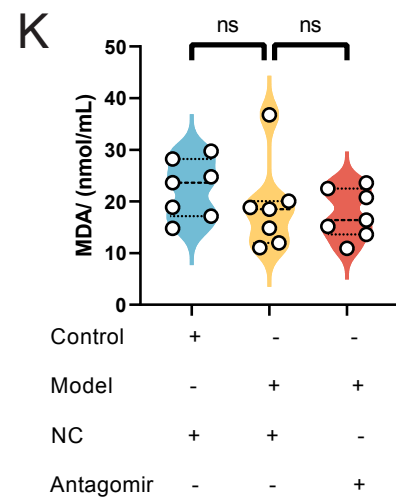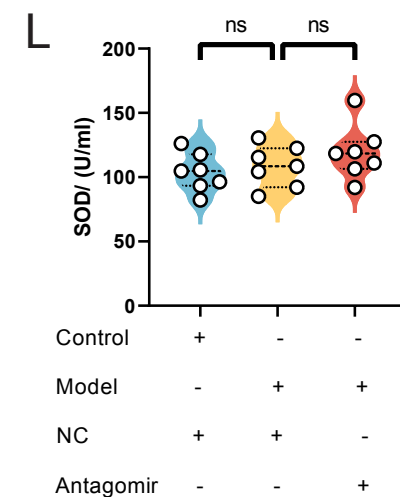

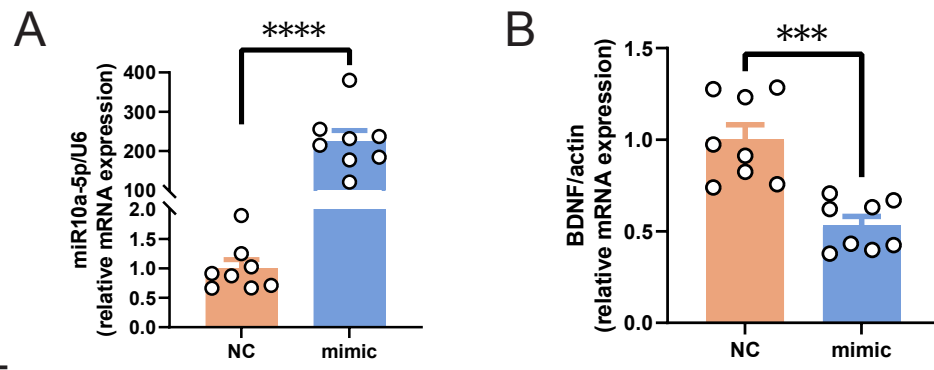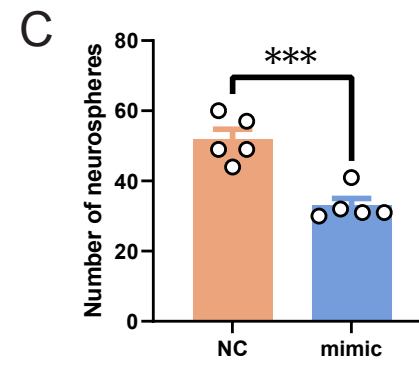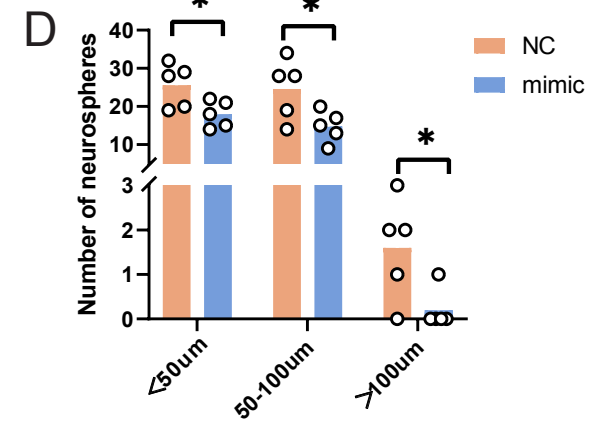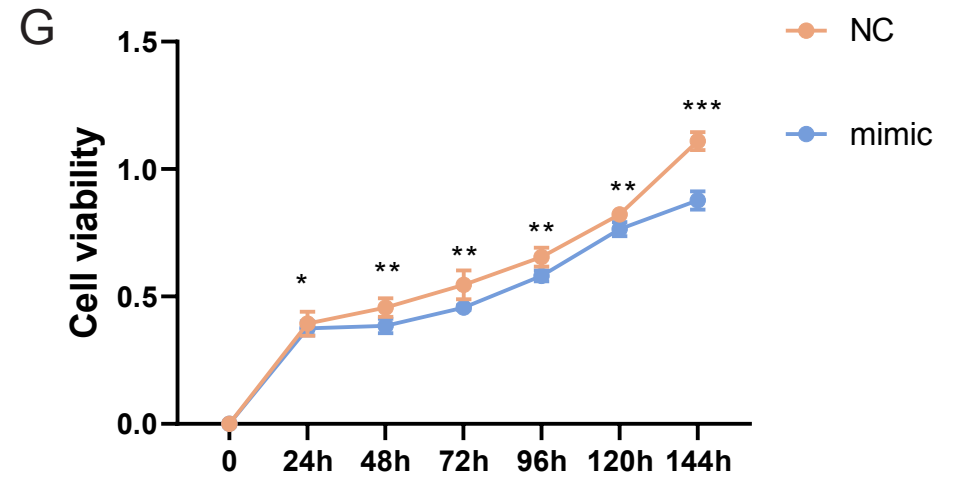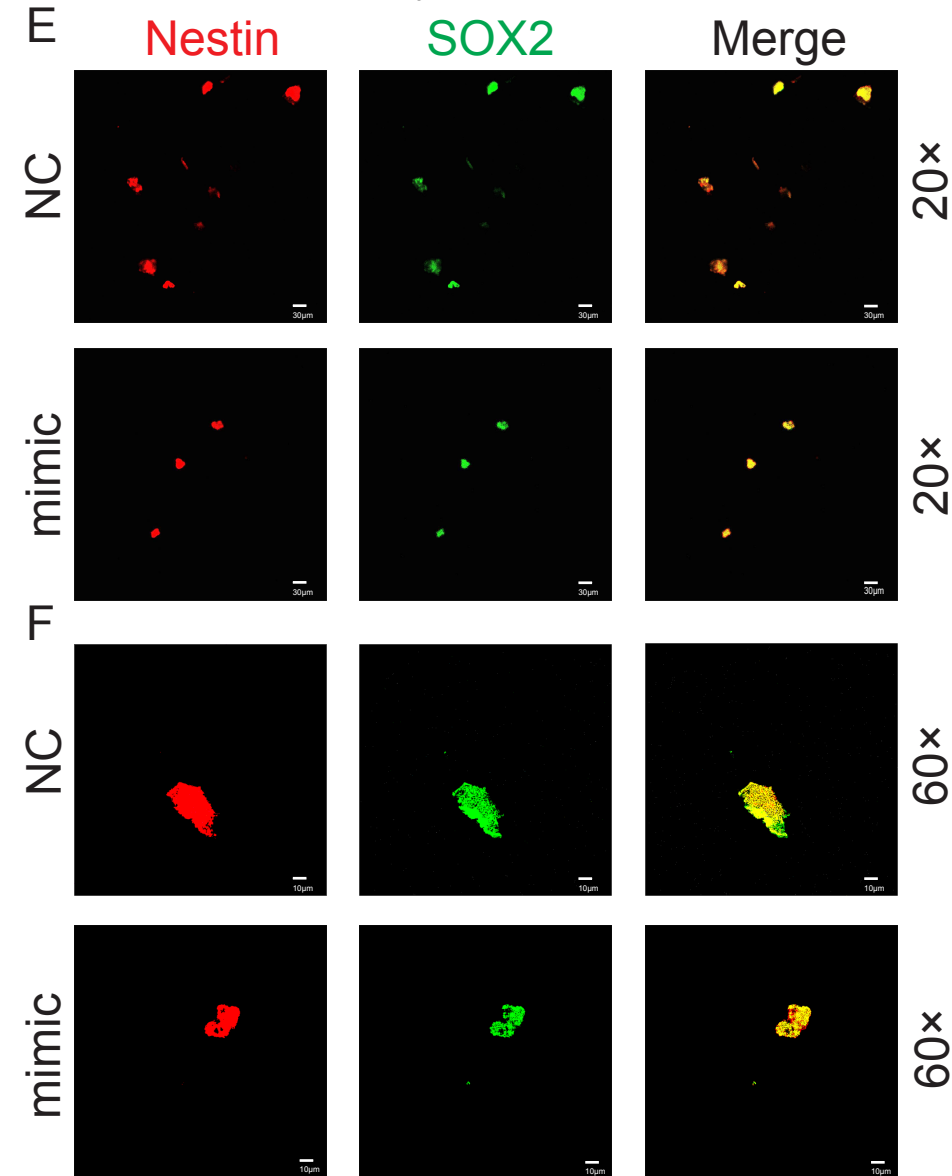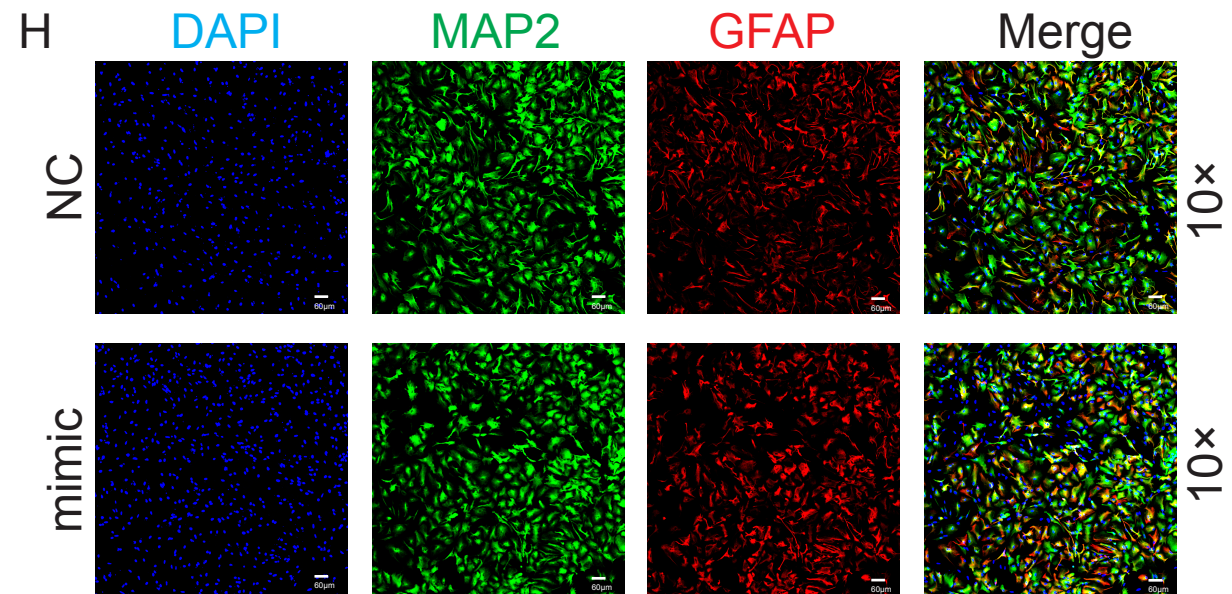

Supplement: Supplementary 1 — Figs. S1 to S5 [file research.0537.f1.zip › Supplement Figures.pdf]
